# The Tweedledum and Tweedledee of dynamic decisions: Discriminating between diffusion decision and accumulator models

**DOI:** 10.3758/s13423-024-02587-0

**Published:** 2024-10-01

**Authors:** Peter D. Kvam

**Affiliations:** The Ohio State University, Columbus, OH USA

**Keywords:** Evidence accumulation, Machine learning, Cognitive modeling, Dynamic choice, Model comparison

## Abstract

Theories of dynamic decision-making are typically built on evidence accumulation, which is modeled using racing accumulators or diffusion models that track a shifting balance of support over time. However, these two types of models are only two special cases of a more general evidence accumulation process where options correspond to directions in an accumulation space. Using this generalized evidence accumulation approach as a starting point, I identify four ways to discriminate between absolute-evidence and relative-evidence models. First, an experimenter can look at the information that decision-makers considered to identify whether there is a filtering of near-zero evidence samples, which is characteristic of a relative-evidence decision rule (e.g., diffusion decision model). Second, an experimenter can disentangle different components of drift rates by manipulating the discriminability of the two response options relative to the stimulus to delineate the balance of evidence from the total amount of evidence. Third, a modeler can use machine learning to classify a set of data according to its generative model. Finally, machine learning can also be used to directly estimate the geometric relationships between choice options. I illustrate these different approaches by applying them to data from an orientation-discrimination task, showing converging conclusions across all four methods in favor of accumulator-based representations of evidence during choice. These tools can clearly delineate absolute-evidence and relative-evidence models, and should be useful for comparing many other types of decision theories.

**Supplementary Information:**

The online version contains supplementary material available at 10.3758/s13423-024-02587-0.

## Introduction

Evidence accumulation models are the gold standard for explaining choice and response times in two-alternative forced-choice [2AFC] tasks, and are widely used in both perceptual (Ratcliff et al., 2016; Heathcote & Matzke, 2022) and value-based choice (Busemeyer et al., 2019) as well as models of neural data (Forstmann et al., 2016). These models are able to shed light on cognitive processes underlying decision-making by using response times, accuracy, and sometimes neuroimaging or process tracing data like mouse-/eye-tracking to make inferences about latent decision processes (e.g., Donkin and Brown, 2018; Gold and Shadlen, 2007; Bogacz et al., 2010; Krajbich and Rangel, 2011).

Typically, evidence accumulation models fall into two categories: (1) *absolute evidence* models, which track the support for each option using distinct accumulators and yield a decision when one reaches a criterion level of support; or (2) *relative evidence* models, which track the balance of support between two options and yield a decision when the balance tips far enough in one direction or the other. For simplicity, I equate the former type of model (absolute evidence) with accumulator models where there are separate, independent processes for separate options (Tillman et al., 2020; Heathcote & Matzke, 2022; Brown & Heathcote, 2008; Smith & Vickers, 1988). The latter (relative evidence) encompasses many types of models including uni-dimensional random walk and diffusion decision models (Diederich & Busemeyer, 2006; Ratcliff, 1978; Ratcliff et al., 2016).^1^ Both types of models include common mechanisms like response caution, information processing speed, non-decision processes, bias, and basic models can be supplemented with parameters that describe attention (Diederich & Busemeyer, 2006; Busemeyer & Townsend, 1993), urgency (Hawkins et al., 2015), learning and memory (Turner, 2019; Miletić et al., 2020; Pedersen et al., 2017), and other processes like decay, loss aversion, or inhibition (Usher & McClelland, 2001, 2004). Likewise, both approaches to modeling dynamic decisions can be driven by a stochastic accumulation process or a deterministic one (Kvam, 2019a).

Although they share many assumptions, there are fundamental differences between absolute-evidence and relative-evidence models that remain unresolved by empirical data. Specifically, relative-evidence models posit that evidence or preference is represented as a balance of support among options, so that support for one option takes away support from the other(s) (Link & Heath, 1975). Conversely, absolute-evidence models like racing accumulators posit that support for any one option does not affect support for another (Brown & Heathcote, 2008; Van Zandt et al., 2000), except in cases where correlations or interactions among accumulators are deliberately introduced (Reynolds et al., 2020; Usher & McClelland, 2001). Absolute- and relative-evidence models therefore make diverging assumptions about the fundamental structure of evidence and preference representations, providing competing theories of latent decision processes.

Despite their apparent fundamental differences, it has proven difficult to identify patterns of data or methods that can discriminate between absolute-evidence approaches like accumulator models and relative-evidence approaches like diffusion decision and random walk models. Indeed, it appears that the two model classes mimic one another’s predictions (Donkin et al., 2011), and account for the same phenomena using similar or even identical mechanisms (Dutilh et al., 2019). The two make differing predictions about what parameters change with practice effects (Heathcote & Hayes, 2012) as well as differing predictions about choice variability when applied to receiver operating characteristic curves in recognition memory (Osth et al., 2017), yet these comparisons are often based on preconceived ideas about what parameters should (not) change with different experimental manipulations. Likewise, model fits to reward rate maximization tasks appear to result in diverging accounts of the effects of stimulus discriminability (Goldfarb et al., 2014). However, the insights in these cases do not tell us much about which model provides a better account per se, but rather tell us which one seems to provide a more sensible interpretation of performance based on a researcher’s beliefs about how its parameters ought to change across experimental manipulations.

An apparent challenge for relative-evidence models like the diffusion decision model is the presence of *magnitude* effects (Pirrone et al., 2022), where response times are faster when a pair of competing choice options are both highly coherent or desirable. The effects appear in both perceptual and value-based choice, indicating that at least some types of decisions and judgments are sensitive to the total support for both options rather than just the balance of support (Turner et al., 2021; Fontanesi et al., 2019). Magnitude sensitivity suggests that the balance of support alone cannot explain empirical patterns of accuracy and response time when the overall desirability or coherence of two competing stimuli is manipulated. However, these manipulations are ultimately inconclusive – a diffusion decision model implementing a relative-evidence decision rule can capture magnitude effects by shifting the noise or diffusion rate associated with high-magnitude trials, meaning that mechanisms in both types of model could account for magnitude effects (Ratcliff et al., 2018).

Much like parallel and serial information processing theories, it may come across as quite difficult to evaluate whether a set of decisions embodies an absolute or relative evidence representation. But also like parallel and serial information processing, these two approaches can and should be distinguished using the right tools (Townsend, 1990). The following sections outline the relationships between relative-evidence and absolute-evidence models, then propose and test several different methods that allow a modeler to determine with a high degree of certainty whether a set of data is best explained by a relative-evidence or absolute-evidence model.

**Fig. 1:**
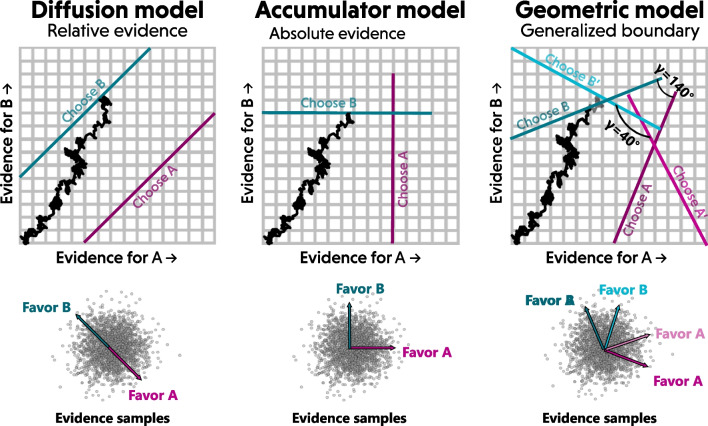
Comparison of stopping rules for diffusion (*left*), accumulator (*middle*), and the generalized geometric form (*right*) of evidence accumulation models. The generalized model allows the choice boundaries to vary freely with the free parameter $\upgamma$, specifying the relative orientation of the two boundaries as shown. The *top row* shows an example accumulation trajectory and where it would terminate for each model, while the *bottom row* shows the evidence distribution (bivariate normal; the same across models) and how it is mapped onto support for each choice alternative (arrows)

### Evidence accumulation

There are at least three components of evidence accumulation models that can differ across approaches: bias or starting point, the sampling and accumulation process itself, and the rules that are used to trigger a response in favor of one option or another. Most of these can be matched across relative-evidence and absolute-evidence models, although individual models may differ in terms of the particulars of how each piece is specified.

For example, in uni-dimensional diffusion and random walk models, evidence accumulation is modeled as a stochastic process, where an overall drift in one direction is complemented by moment-to-moment fluctuations in evidence strength (Ratcliff 1978, 2018; Itô 1974). This is the case in some accumulator models (Smith & Vickers, 1988; Hawkins & Heathcote, 2021; Anders et al., 2016; Matzke & Wagenmakers, 2009), but there are other accumulator models where evidence accumulation is deterministic and/or ballistic (Brown & Heathcote, 2005, 2008; Kvam et al., 2023). These models still typically incorporate a drift rate that controls the rate of evidence accumulation and across-trial variability in drift, but do not incorporate the diffusion process that inserts noise into the accumulation process.

In general, the deterministic accumulation process proposed by models like the LBA can be seen as a special case of the more general stochastic accumulation process, but with the diffusion rate $\upsigma$ set to 0 rather than customarily set to .1 or 1 (Donkin et al., 2009). However, this raises important considerations related to model falsifiability, as the distributions of drift rates can take (and be mimicked by) many alternative forms (Jones & Dzhafarov, 2014). For simplicity and to sidestep this concern, this paper focuses on the general case where evidence accumulation is stochastic, if only to put all the models considered here on equal footing in terms of their flexibility. Likewise, it does not focus on accumulator models that have interactions or correlations between accumulators (Usher & McClelland, 2001; Reynolds et al., 2020). However, analyses of the Leaky Competing Accumulator model and the Linear Ballistic Accumulator model are provided in the supplement, and code for both models is provided on the corresponding OSF page at osf.io/ctz37.

With this issue resolved, one can equate the evidence accumulation process between relative-evidence and absolute-evidence models. A more formal mathematical characterization is given in Appendix A, but it is helpful to provide an overview here. Samples of evidence in a relative-evidence diffusion decision model are normally distributed, with a mean $\mu$ given by the drift rate and variance $\upsigma ^2$ given by the diffusion rate. Samples of evidence in an absolute-evidence accumulator (racing diffusion) model are bivariate normally distributed, with the mean of the bivariate normal given by drift rates $\mu _A$ (x) and $\mu _B$ (y) and symmetric variance given by the diffusion rate $\upsigma ^2$.

As I show in the next section, the underlying distribution of evidence can actually be considered to follow a bivariate-normally distribution (see bottom row of Fig. 1). The mapping of this state onto support for each option is the key distinguishing feature of relative-evidence and absolute-evidence models. In a relative-evidence model, one can take a“slice” of the evidence distribution, as shown at the bottom left of Fig. 1, mapping it onto a single dimension. This results in a uni-dimensional accumulation process, where evidence for one option is commensurate with evidence against the other option. The two options are opposites, represented as two ends of a single continuum.

In an accumulator model, the support a piece of evidence provides for each option can be found by instead taking a horizontal (A) or vertical (B) “slice” of the evidence distribution, or simply the marginal distributions along the x and y dimensions. This slides will again be normally distributed when projected onto either dimension because the underlying bivariate normal distribution of evidence from which accumulator values are derived is radially symmetric.

As a result, the support or balance of support for each option at each time step is consistently normally distributed, whether working with a diffusion model, accumulator model, relative-evidence, or absolute-evidence. This makes for relatively easy comparisons among the different approaches to modeling evidence accumulation.

#### Start points

So far, the absolute-evidence and relative-evidence processes covered have assumed a balanced initial point – in Fig. 1, this corresponds to the origin [0,0]. In doing so, I have largely ignored the role of start points in the evidence accumulation process. This is not an essential component of many models, as demonstrated by Tillman et al. (2020), but it does help account for processes like fast errors (Ratcliff & Rouder, 1998; Brown & Heathcote, 2008). Differences in start points also reflect biases toward one option or another, due to things like prior probabilities, differences in rewards for selecting one option over another, or differences in desired certainty between the two options (Puri et al., 2023; Diederich & Busemeyer, 2006).

As with drift rates, accumulator models typically assign a different start point to each option (Puri et al., 2023), either as a fixed value or a draw from a distribution of start points (Brown & Heathcote, 2008). Thus, even if there is no bias, there may be some variability in the start point of each accumulator. The distribution of start points in an accumulator model is commonly modeled as a uniform distribution (Brown & Heathcote, 2008) sampled separately for each accumulator. Conversely, the diffusion decision model assumes a uniform distribution of starting points over the balance of evidence ($A-B$) (Ratcliff & Rouder, 1998; Ratcliff & Smith, 2004). In terms of support for individual options, a uniform distribution across the difference implies non-independence of starting points across choice options.

For example, a uniform start point on the balance of support $A-B$ would appear in Fig. 1 as a region bounded by two parallel diagonal lines $y = x \pm s_v$, where $s_v$ is the variability in start points. Conversely, a uniform distribution of start points for each of the accumulators separately would appear as a box bounded horizontally by $y = s_v$ and vertically by $x = s_v$ (also assuming they are lower bounded by x = 0 and y = 0 Kvam et al., 2023). Projected onto the $v_A$-$v_B$ axis, this would no longer be uniform. For example there are many more combinations of the two uniform random variables $s_{v_A}$ and $s_{v_B}$ that could result in a balance of zero, while only a single combination of $s_{v_A}$ and $s_{v_B}$o could result in a start point equal to $+s_v$ ($s_{v_A} = s_v$ and $s_{v_B} = 0$).

The influence of these differences in start points depends entirely on how great the start point variability is compared to the thresholds in the model. With high start point variability and low thresholds, these start points will have a substantial impact on model predictions. With low start point variability and high thresholds, their impact will be minimal. Because models do not agree on the shape of start point distributions, this paper focuses mainly on cases where both options start with no evidence, to ensure that they are matched as closely as possible. In the model comparison of the Application section I show below, I allow both models to implement their own versions of start point variability. The same analyses presented here can be carried out for different combinations and assumptions about the starting points of an evidence accumulation process as well, allowing modelers to directly compare uni-dimensional diffusion / random walk models, accumulator models, and the generalized version where $\upgamma$ is a free parameter.

### Generalized geometric model

As Fig. 1 hints, absolute- and relative-evidence models are not a dichotomy, but rather two points along a continuum. Models of the kind described above are both special cases of a more general geometric representation of the evidence accumulation process (Kvam, 2019a). Specifically, a diffusion decision model represents the evidence accumulation process such that the options are represented as opposing directions: evidence for option A (e.g., up) is evidence against option B (e.g., down) and vice versa. If A and B are the only two options, this allows a decision-maker to represent the evidence state as a balance between them (Ratcliff et al., 2016). Information favoring one option tips the balance in the corresponding direction, in a zero-sum competition between choice options. Such a representation is optimal for two option choice, as the balance of support is directly proportional to the relative log odds between the two options allowing a decision maker to stop when the probability of one option being correct exceeds the criterion or threshold value (Wald & Wolfowitz, 1949; Bogacz, 2007).

Spatially, the degree of support for option A in a uni-dimensional random walk or diffusion model should be 180 degrees away from the degree of support for option B, so that they are in direct conflict with one another. The balance of support is then the project of the state of the evidence accumulation process onto the A-B axis, and the thresholds for choosing option A and option B will appear as parallel lines. This arrangement is illustrated in the left panel Fig. 1.

A typical accumulator model represents evidence for two options, A and B, separately and independently (Heathcote & Matzke, 2022). In terms of a spatial orientation or arrangement, support for the two options can be represented as perpendicular spatial dimensions. For example, the *x* coordinate of an accumulator in two dimensions might correspond to the degree of support for option A, and the *y* coordinate might correspond to the degree of support for option B. As a consequence, changes in support for one option do not affect the degree of support for the other option. This is illustrated in the middle panel of Fig. 1. The thresholds for choosing option A and option B should also be perpendicular, so that moving far enough in the *x*-direction triggers a choice in favor of A and moving far enough in the *y*-direction triggers a choice in favor of B.^2^

The two options being oriented at 180 or 90 degrees relative to one another, respectively corresponding to relative-evidence and absolute-evidence models, only account for two out of a wide range of models we can design when thinking spatially. In fact, we can think of the angle between choice options as a free parameter $\upgamma$, such that $\upgamma = 180$ degrees is a relative choice boundary like the thresholds of a diffusion model, and $\upgamma = 90$ degrees is an absolute choice boundary like the thresholds in an accumulator model. Theoretically, $\upgamma$ describes the similarity between choice options and in principle can vary from 0 (choice options are indistinguishable) to 180 degrees (choice options are polar opposites) (Kvam, 2019a; Kvam et al., 2023). A few other possibilities are shown in the right panel of Fig. 1: a value of $\upgamma = 40$ yields choice boundaries oriented in almost the same direction, while a value of $\upgamma = 140$ yields choice boundaries somewhere between absolute-evidence and relative-evidence models.^3^

Formally, the degree of support a piece of evidence provides for a choice option is computed by projecting a vector describing the piece of evidence onto the vector describing a choice alternative (Kvam, 2019a; Kvam & Turner, 2021). Details are presented in Appendix A, but essentially, we can take the current state or the distribution of evidence samples and calculate its component in the direction corresponding to each option under consideration. When enough support for an option has been obtained (i.e., the component exceeds a critical value $\uptheta$), it is chosen over the others – just as in the relative-evidence and absolute-evidence approaches.

Different values of $$\upgamma $$ can be used when the choice options share similarities or fall along a continuum. For example, Kvam and Busemeyer (2020) represented price responses on a pricing task such that similar dollar values (e.g., $1 and $2) had very small values of $$\upgamma $$, while larger differences among dollar values (e.g., $0 and $20, the maximum participants could receive in the experiment) had larger values of $$\upgamma $$ and thus larger angles between them. Along the same lines, similar colors or stimulus magnitudes can be represented by converging spatial directions (small $$\upgamma $$) and distinct or dissimilar ones can be represented by diverging directions (large $$\upgamma $$) (Kvam et al., 2023).

The exact value of $\upgamma$ for a pair of choice options is an empirical question, and it can be estimated based on the patterns of responses and response times for a given task or choice pair. However, it should correspond to real relationships between choice options. That is, the more closely related two options are – or the more similar their neural representations (Kriegeskorte et al., 2008) – the smaller $\upgamma$ should be. In some cases, it can be estimated independently from additional data like similarity ratings (see Study 2 in Kvam et al., 2023). In other cases, it directly describes a conceptual association or similarity that is key to performance, such as response time and accuracy on the Implicit Association Test arising from the associations between concepts or categories presented (Kvam et al., 2024). Although only a single value is used here because we are presently concerned with binary choice, the relative orientations of choice options correspond to psychologically meaningful relationships among their representations, and are critical to understanding multi-alternative choice (Kvam, 2019a; Kvam et al., 2023).

In the Machine Learning and Application sections, I show how $\upgamma$ can be directly estimated from the accuracy and RT data. This allows estimates of a free parameter in the geometric approach to index how closely a decision process can be approximated by a relative-evidence or absolute-evidence model.

## Discriminating between models

With so many similarities between relative-evidence and absolute-evidence models, it should be no surprise that they often show a high degree of mimicry (Donkin et al., 2011). Perhaps a more difficult question is whether, when, and how one can tell the difference between the dueling evidence accumulation models. It will not be apparent which approach is better, which should be used for an analysis, or even if there is value in having a general case unless the models make differentiable predictions. To make the addition of the $\upgamma$ parameter and the differentiation shown in Fig. 1 useful, we must be able to identify what (if any) differences there are between absolute-evidence and relative-evidence model predictions for accuracy and response times.

In this section, I describe four ways that relative-evidence and absolute-evidence models can be distinguished from one another. The first takes a look at the stopping rules implemented in each model and reflects on their effect on the conditional distributions of evidence that a decision-maker might collect if they were implementing different strategies; the second one examines all of the factors that go into drift rates and how these factors are delineated in different modeling approaches; the third uses a machine learning classifier to directly quantify the posterior probabilities that a given set of data was generated by either diffusion or an accumulator models; and the final method uses machine learning to precisely estimate $\upgamma$ in a more general framework.

### Accumulated-evidence profiles

The first way to discriminate between absolute-evidence and relative-evidence models is to look at what information a decision-maker considered en route to their choice. Because uni-dimensional random walk models focus on the *balance* of support between options, a decision is typically made when the decision-maker samples a piece of evidence that strongly favors one option at the expense of another. In absolute-evidence models, information can favor both responses simultaneously because there is no direct conflict between them. In these cases, the net balance of support does not change, yet positive information favoring all options pushes an accumulator model closer to the decision boundary by incrementing the support for each one. This difference is at the root of why magnitude effects are thought to provide support for an accumulator-based framework – increasing the value or magnitude of both options simultaneously can result in faster RTs even when the difference between them remains unchanged (Kirkpatrick et al., 2021) which makes little sense from the standpoint of relative-evidence models.

Because uni-dimensional random walk and diffusion decision models only track the balance of support, samples of evidence that favor both options (or neither option) are unlikely to carry the evidence state across a decision boundary, and thus ambivalent or moderate samples are unlikely to terminate a decision process driven by the balance of evidence. As a result, moderate evidence – which only weakly favors one option over another – tends to be under-sampled in decision processes drive by relative evidence relative to strong evidence that heavily tips the balance toward one option or the other (Kvam et al., 2022). As a result, the last pieces of evidence someone collects before making a decision is more likely to be a sequence of unusually strong or extreme evidence than a run of moderate or weak evidence. In uni-dimensional random walk models, therefore, there should be an oversampling of extreme evidence that tips the balance of evidence far in one direction, as well as an undersampling of moderate or ambivalent information that favors both options.

**Fig. 2:**
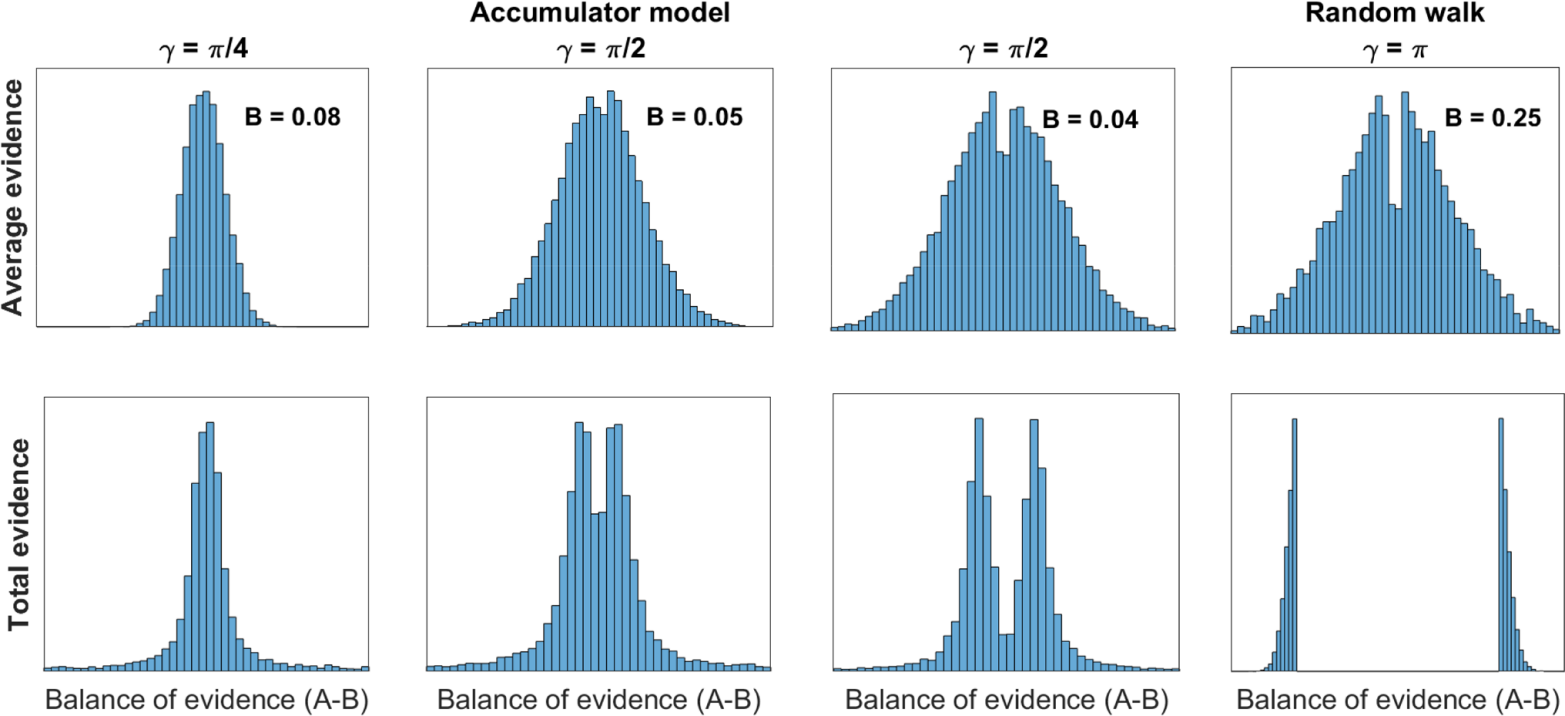
Changes in the expected distribution of evidence as a function of the angle between choice alternatives. The *top panels* show the expected distribution of average/normalized evidence (balance of support divided by number of samples) that a decision maker might have accumulated at the end of a trial. The *bottom panels* show the cumulative evidence that a decision maker might have accumulated at the end of a trial (no normalization by number of samples). Each column corresponds to a different choice strategy, described as different angles $\upgamma$ between choice options. Values of $\upgamma = \frac{\uppi }{2}$ and $\upgamma = \uppi$ correspond to accumulator and uni-dimensional random walk models, respectively

Absolute-evidence accumulator models, by contrast, do not depend on this balance of evidence and as a result do not yield the same under-sampling of moderate evidence. Instead, a decision-maker who uses an absolute-evidence representation will tend to draw a relatively representative distribution of evidence as they make their choices. Ambivalent information adds to both accumulators, meaning its effect on support for one option does not cancel out the support it confers to the other option, but rather is integrated as an increment to both accumulators. As a consequence, ambivalent information is no less likely to produce a decision than evidence that favors only one option. This creates an evidence distribution that mimics more closely the properties of the stimulus, reflecting well the input information that a decision-maker considers during choice. It is still not entirely free from bias, however, as an extreme sample of evidence is still more likely to produce a response than a moderate or weak sample – meaning that the final sample producing a response is likely to be extreme rather than moderate. This slightly reduces the proportion of moderate information sampled even in an absolute-evidence, independent-accumulators model.

These different patterns of evidence collection can be detected by looking at the sum or average of the information presented to a participant, as shown in Fig. 2. For uni-dimensional random walk / relative evidence models, the under-sampling of moderate and ambivalent evidence will appear as a gap in the middle of the distribution, showing the relative lack of ambiguous information that a decision-maker collects. As a result, the average and total difference in evidence for option A and option B will appear bimodal. Furthermore, this distribution will have greater variance than the true distribution of evidence provided by the stimulus (Kvam et al., 2022), as extreme information is over-sampled relative to the true distribution of information provided by the stimulus.

By contrast, an absolute-evidence accumulator model is shown in the second panel from the left in Fig. 2. This model ends up generating a balance of evidence that relatively closely approximates the true underlying stimulus distribution. A decision is still slightly more likely to be triggered by a strong piece of evidence for option A or for option B, resulting in the smaller gap in the balance of evidence at the end of the choice process (as shown in the bottom panel). This effect is much weaker than for uni-dimensional random walk models, as moderate information or information that does not favor one option over the other – provided it provides some support for both options – can still trigger a decision. As a result, one should not expect substantially inflated variance in the distribution of accumulated evidence in an accumulator-based model (Kvam et al., 2022) relative to the variance of information randomly sampled from the stimulus.

I refer to these predictions as *accumulated-evidence profiles*, describing the patterns in accumulated evidence that decision-makers collect en route to a choice. These connect the goal of the decision process – either to identify a good option (absolute evidence) or the better option (relative evidence) – to the pattern of information search it yields. As a result of these diverging predictions, it should in principle be possible to identify which type of model generated a set of data by looking at the stimulus information a participant collected. One need only compare the set of evidence considered by a decision-maker against the patterns predicted by each model. This can be done using distribution-based metrics like K-L divergence, or by generating likelihood functions for expected distributions of evidence from each of the models using approaches like probability density approximation (Turner & Sederberg, 2014; Holmes, 2015). Once this is done, the generative model can be identified using model comparison metrics (Wagenmakers et al., 2010; Myung, 2000) or by using them in model classification approaches like the ones I describe in the machine learning section below.

In the most basic version of accumulated-evidence profiles, the representation of evidence directly corresponds to samples from the true stimulus or environment. However, psychological representations do not always directly reflect the physical properties of a stimulus (Shepard, 1962). Accumulated-evidence profiles do not require this, but they are most effective when true information from the stimulus is proportional to the strength it carries in changing beliefs. That is, as long as moderate stimulus information corresponds to moderate evidence and strong stimulus information corresponds to strong evidence, the predicted patterns of evidence shown in Fig. 2 should hold.

#### The continuous case

The paper by Kvam et al. (2022) focused on discrete-sample random walks, where a decision-maker considers information piece by piece. However, diffusion (as opposed to random walk) models are typically concerned with a continuous information sampling process. In these models, the instantaneous evidence accumulation follows a Brownian motion process. Here, I extend the proof from the random walk to the continuous diffusion decision model.

Fortunately, the same finding applies to the continuous case. Over time, the position of the stochastic diffusion process results in a state that follows a normal distribution. Taking the expected distribution of evidence states at any given point in time and normalizing that distribution by the amount of time has passed results in the same distribution no matter how much time has passed. For example, if a process with drift $\mu$ and noise $\upsigma ^2$ runs for $t=2$ seconds, it will be normally distributed with mean $2\mu$ and variance $2 \upsigma ^2$. If it runs for $t=4$ seconds, it will be normally distributed with $4\mu$ and variance $4 \upsigma ^2$. As a result, the “true” distribution of evidence or information from the environment, divided by the amount of time the stochastic process has run, will continue to be normally distributed.

The key element of a diffusion decision model that creates a biased sample of evidence is the stopping rule, specified by the threshold $\uptheta$. Because the steps in a diffusion process are infinitesimal, a decision-maker implementing a diffusion decision model to make a choice will always stop at exactly $\pm \uptheta$ as the balance of evidence. Therefore, to calculate the normalized distribution of evidence at the time of choice, we need only take $\uptheta$ and divide it by the response times for correct decisions, and take $-\uptheta$ divide it by the response times for incorrect decisions. This is then weighted by the relative frequency (probability density) of observing that balance of evidence / response time. Formally, the probability of observing a balance of evidence $\frac{\uptheta }{t}$ is the probability density of the diffusion decision model $f(t | \uptheta , \updelta , \upbeta , \tau , \upsigma ^2)$ where $\uptheta$ is the threshold, $\updelta$ is the drift rate, $\upbeta$ is the bias / start point, $\tau$ is the non-decision time, and $\upsigma ^2$ is the noise / diffusion rate. Naturally, this would have to be integrated over drift rates or start points when trial-to-trial variability in these parameters is introduced (Ratcliff, 1978; Ratcliff & Rouder, 1998).

**Fig. 3:**
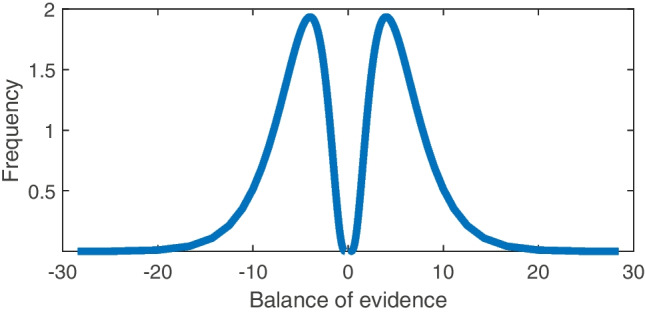
Distribution of accumulated evidence obtained from a diffusion decision model, using values of threshold $\uptheta = 1$, drift $\mu =.5$, noise $\upsigma ^2 = 1$ and no bias or non-decision time

The resulting distribution of evidence – computed by evaluating the likelihood of obtaining $\frac{\uptheta }{t}$ for many values of *t* – is shown in Fig. 3. As illustrated, the distribution of evidence is still starkly bimodal. This indicates that it is not the discrete sampling of evidence or overshooting of the decision boundary that produces polarized samples of evidence, but rather the stopping rule of relative-evidence models (encompassing the DDM) that filters out evidence states near zero.

In short, a diffusion decision model or any relative-evidence model predicts a bimodal distribution of evidence at the end of a trial. In many cases, these models constitute optimal strategies for information sampling that minimize response times for a desired level of accuracy (Bogacz et al., 2006), meaning that a “rational” person should collect information in this way. As a result, anyone who implements the optimal strategy for information sampling will be led to a polarized, bimodal distribution of evidence, deepening the divide between people (even optimal or rational agents) who reach opposing decisions.

#### Limitations

Despite being apparently straightforward, there are a few caveats that limit the use of accumulated-evidence profiles for practical applications. The first is that many perceptual decision-making studies do not record or cannot track exactly what a participant sees during every trial. For dynamic stimuli (Ball & Sekuler, 1987; Shadlen & Newsome, 2001), the frame-by-frame information that a participant is shown is often not recorded or difficult to recover without the random seed used to generate them during an experiment (Pleskac et al., 2022). In other cases, it is hard to say what exactly a participant saw, either because there is more information on the screen than a participant can attend, because visual fixations or inspections are not recorded, or because there are multiple stimuli or attributes within a foveated area (Pleskac et al., 2019).

For cases where the stimulus information and the information that participants saw is accessible, it is possible in principle to form accumulated-evidence profiles. However, these profiles may actually consist of two “phases” of evidence accumulation: pre-decision evidence and post-decision evidence (Pleskac & Busemeyer, 2010). Pre-decision evidence consists of everything that actually entered into a choice and should give us insight into whether a person was using a relative or absolute evidence strategy. Post-decision evidence can arrive after a decision maker has chosen, during the period after they have triggered a decision and when they have actually completed the motor action to enter it (Weindel et al., 2022). This post-decision evidence will consist of random draws from the stimulus, meaning that the distribution of evidence at the end of a trial will be a mixture of random samples (post-decision stimulus) and nonrandom samples (pre-decision stimulus) that are biased by the stopping rule.

The time between a decision and the motor response can be a serious problem when the stimulus has frequent updates. For example, a dynamic stimulus that refreshes at 60Hz might have 12 additional random draws if a participant takes a (fairly minimal) 200ms to enter their response. Because we do not know exactly when the decision is truly made nor how long non-decision time is, it is difficult to evaluate exactly how contaminated each accumulated-evidence profile is with random stimulus information. This might be rectified in the future by examining neural signatures of decision-making (Gold & Shadlen, 2007) to identify more exact decision times, at which point the accumulated-evidence profiles could be truncated more closely to the “true” decision time.

At present, this type of analysis seems limited to very specific experimental paradigms. However, I examine how well accumulated-evidence profiles can be used to identify generative models and how well it aligns with other methods of discriminating between relative-evidence and absolute-evidence models in the Application section. At minimum, it is clear from these simulations that relative-evidence and absolute-evidence models posit stopping rules that result in diverging patterns of information search, and as a result, very different accumulated-evidence profiles.

Accumulated-evidence profiles can in other cases be seen as a supplementary piece of information on how participants are deciding, which can be used alongside response times and accuracy to determine what model provides the best account of behavior. Finding a bimodal distribution of accumulated evidence might help make it clear that a particular individual was using a relative-evidence approach to make choices, even when the response time distributions or accuracy are not conclusive. Therefore, even with their limitations, accumulated-evidence profiles can shed some light onto the generative processes underlying dynamic decisions.

### Disentangling drift

A key element of both absolute-evidence and relative-evidence models is their account of how beliefs or preferences change over time. Both types of models include a *drift rate* describing the average rate at which a person shifts toward one option, either individually or in relation to the other (see Fig. 1). This drift rate encompasses a wide range of information about both the decision-maker and the stimulus, ranging from the clarify of the stimulus, the degree of attention the decision-maker is paying, and many other aspects that enter into the signal-to-noise ratio during evidence accumulation (Ratcliff et al., 2016). There are several different components of the stimulus, decision maker, and choice options that are useful to examine separately, dissecting the overall drift rate into constituent parts. Specifically, we can disentangle drift into at least three meaningful elements: *coherence*, describing how clear the information provided by the stimulus is; *match*, describing the degree to which a decision maker’s beliefs or preferences about the stimuli align with the choice options they have; and *discriminability*, which describes how easy it is to tell the difference between the options that decision maker has in principle.

In most applications, manipulations of coherence, match, and discriminability are described by changes in the drift rates of the evidence accumulation process. However, random walk models were influenced heavily by the sequential probability ratio test, which uses a log-odds representation such that the balance of evidence can be mapped onto a probability of one option being correct (Wald & Wolfowitz, 1949; Bogacz et al., 2006; Smith & Ratcliff, 2015) in inferential choice situations. To the extent that relative evidence models follow this original design, we can examine how coherence, match, and discriminability map onto the degree of support for each option.

For coherence and match, the relationship to drift rates is extremely straightforward. The drift rate for the correct option should increase linearly as coherence increases, directly indexing the signal-to-noise ratio (Eckhoff et al., 2008; Lee & Usher, 2023). Likewise, the drift rate should increase with the degree of match, reflecting a shift in the proportion of samples that favor one option over the other (Diederich & Busemeyer, 2003).

Discriminability should be related to drift rates in a relative evidence diffusion model. The key piece that links the diffusion model to optimal inference is the fact that the balance of evidence is linearly related to the log odds of one option relative to the other in two-alternative choice (Wald & Wolfowitz, 1949; Bogacz et al., 2006; Bogacz, 2007; Kvam & Pleskac, 2016). This means that the balance of evidence should be directly related to the discriminability of the two choice options, such that more discriminable options have a stronger balance of evidence given the same stimulus information.

To make this clearer, it helps to consider an example. Imagine a decision maker is pulling balls (with replacement) from an urn, and is tasked with using the balls to determine whether the urn is filled with more blue balls or more red balls. Specifically, the decision might be between 55% blue / 45% red balls or 45% blue / 55% red balls. This is a relatively difficult decision because the two choice options are similar to one another. Assuming equal prior probabilities, drawing two red balls in a row (D) gives a posterior probability of the red urn (R) as

1 $$\begin{aligned} Pr(R | D) = \frac{Pr(D|R)\cdot Pr(R)}{Pr(D)} = \frac{(.55)^2 \cdot (.5)}{(.55)^2 \cdot (.5) + (.45)^2 \cdot (.5)} \end{aligned}$$

This works out to be slightly less than 60%. We can compare this to the case where the competing urns are easier to tell apart, such as 90% blue / 10% red versus 10% blue / 90% red. In this case, the probability of the urn having more red balls is $\frac{(.9)^2 \cdot (.5)}{(.9)^2 \cdot (.5) + (.1)^2 \cdot (.5)}$, or nearly 99%. For the same information, the posterior odds of two highly discriminable choice options (90/10) will be much more extreme than for options that are hard to discriminate (55/45) (Edwards, 1965).

This is a roundabout way to say that the drift rate in a uni-dimensional random walk or diffusion model should increase with coherence, match, and discriminability. This should imply that response times decrease and accuracy increases as any of the three are titrated. However, discriminability is slightly different than coherence and match in that it may be known before the stimulus appears on screen. This means that a decision-maker can actually adjust their degree of response caution in response to a pair of choice options anytime they see the choice options appear before the stimulus. In some cases, this can lead to increases in both accuracy and response times, corresponding to higher thresholds in the model.

Overall, we should either expect accuracy to increase while response time decreases (drift rate change) or both accuracy and response times to vary in the same direction (speed-accuracy trade-off) as discriminability increases in a uni-dimensional random walk or diffusion model with a relative-evidence decision rule. A pattern where accuracy fails to increase with this manipulation, or where accuracy and response time do not closely covary, would be inconsistent with such a model.

#### Absolute-evidence models

Absolute-evidence approaches like accumulator models should logically make similar predictions for match and coherence. Increased match would lead to a greater drift rate for the favored option and lower drift rate for the disfavored option, on average yielding faster and more accurate responses for higher levels of match. Likewise, increasing stimulus coherence should result in higher drift for the correct choice option and lower drift for the incorrect choice option. In these predictions, accumulator models do not differ from diffusion models

However, it is not clear that accumulator models should make the same predictions for discriminability, as accumulator models are not derived from the same log-odds representation of evidence (Vickers, 1970; Smith and Vickers, 1988; Reynolds et al., 2020, although in some models of confidence the two have been linked;). There is nothing “built in” to accumulator models that reflect the discriminability of the choice options, as the representations of support for the two are typically kept separate. As a result, more discriminable choice options may not lead to any change in choice response times or accuracy at all because response times are conditional only on the winning accumulator. As with diffusion models, shifts in thresholds may produce faster response times and lower accuracy (lower thresholds) or slower response times and greater accuracy (higher thresholds); yet it is difficult to derive strong predictions for how drift rates might vary with discriminability like the relative evidence diffusion models do.

In the Application section of this paper, I present the results of an experiment that manipulated discriminability alongside match and coherence. To preview the results, the manipulation of discriminability has little effect on response times while leading to greater accuracy.

#### Separate model mechanisms

Zooming out for a moment, neither modeling approach is really all that clear about how coherence, match, and discriminability must affect the decision process. There is nothing in a diffusion decision model or accumulator model that requires that these are the only ways the models can account for the three types of manipulations. In fact, they each might deal with the effects of these manipulations empirically by varying drift, thresholds (e.g., for discriminability that is known prior to stimulus onset), or even other parameters like noise or drift variability. The problem is that “drift” encompasses such a wide range of stimulus and choice-option manipulations that it is hard to interpret exactly what a shift in drift rate means.

I point this out not to undermine the predictions derived above, but to emphasize that there is a lack of clarity around different types of manipulations and their relationship to model parameters. The effect of discriminability comes from drawing relative-evidence and absolute-evidence models as far as possible toward their logical conclusions. These are not necessarily essential predictions of either approach, but they are as near as one might expect to their canonical responses to manipulations of discriminability.

This is a key point where the geometric approach becomes deeply informative relative to the special cases of absolute- and relative-evidence. Because the evidence accumulation process is multidimensional and the response options are described separately from the stimulus information, we can actually isolate the effects of coherence, match, and discriminability to separate parameters.

Specifically, coherence should affect the signal-to-noise ratio of the accumulation process, which is fundamentally what it does. Increased coherence should correspond to a higher drift magnitude $|\mu |$, i.e., a stronger signal driving evidence accumulation. This is the closest to a drift rate in the generalized model, and the most straightforward stimulus manipulation. Greater coherence will naturally result in greater accuracy and faster response times, regardless of what option is favored.

Next, match should determine the drift *direction* $\upphi$. The direction of accumulation, or central tendency of the stimulus, affects the rate of evidence accumulation only insofar as it aligns better or worse with one of the choice options. Changing the central tendency of a stimulus distribution does not inherently make a decision easier or harder on its own, meaning it shouldn’t affect the drift rate / magnitude directly. Rather, manipulating the stimulus so that it better aligns with one of the options *v* increases the component of the drift along that option, comp$_v(\mu )$. In the geometric view, this makes it conceptually and empirically distinct from coherence in that it affects a different part of the accumulation process. In more formal terms, they are actually affecting different dimensions of evidence accumulation: in polar coordinates, coherence affects the radius of the evidence distribution while match affects the angle (Smith, 2016; Kvam, 2019a).

Finally, discriminability directly corresponds to the parameter $\upgamma$ that I introduced in the introduction. How different the options are inherently determines the ease with which someone can tell them apart, tying discriminability to the angle between response options. In this view, discriminability does not affect anything to do with the stimulus representation or evidence accumulation state; it only affects the relationships between the two options. However, this has downstream implications for stopping rules. When two options hard harder to tell apart (less discriminable / lower $\upgamma$), a decision-maker must gather more evidence than when they are easier to tell apart (more discriminable / higher $\upgamma$). As a result, a decision-maker is likely to be less accurate if their choice boundaries are the same. Recognizing this, they may increase their thresholds $\uptheta$, slowing down the decision process in an effort to maintain accuracy. This possibility was thoroughly explored in recent work on multi-alternative choice by Kvam et al. (2023), which validated this prediction.

The geometric approach therefore delivers a piece that is missing from traditional evidence accumulation models, which is a delineation of the effects of signal strength, signal-option match, and option discriminability. Empirically, this allows such a model to perform better (Kvam et al., in press), but the conceptual and theoretical benefit is surely there as well.

### Machine learning

In addition to accumulated-evidence profiles and manipulations of discriminability, there are at least two other ways in which we might discriminate between relative-evidence and absolute-evidence models. Both of the final methods are based on a machine learning approach that uses deep neural networks to make inferences about generative models from the observable response time and accuracy data. The two approaches have complimentary goals: one seeks to directly discriminate between models, while the other seeks to connect them to the more general GSR framework outlined above in order to evaluate the relationships between choice options.

The first *classification* approach involves training a neural network to classify a set of accuracy and response time data according to which model was used to generate it Radev et al. (2021). This allows us to take behavioral data from an individual or group of participants and determine whether a diffusion or accumulator model provides a better account or closer match to the true data. Formally, it permits assigning a posterior probability to each model on the basis of the observed data – approximating formal Bayesian model comparison similar to Bayes factors (Rouder et al., 2018).

Second, I examine an *estimation* approach, where I directly estimate a parameter $\upgamma$ that describes the relationship between choice alternatives, as shown in Fig. 1. This takes the generalized view of evidence accumulation described at the start of this paper (Kvam, 2019a; Kvam & Turner, 2021) , where relative-evidence and absolute-evidence models are just two points on a continuum of possible representations that participants might be using to make their decisions. The estimation approach also simultaneously provides estimates of other model parameters, including drift rates, drift direction, thresholds, non-decision time, and drift rate variability. Note that these are all estimated as free parameters – while previous approaches have noted some difficult in simultaneously estimating all of these (Diederich & Mallahi-Karai, 2018), Fig. 5 shows that it is possible to do using the machine learning method.

#### Classification approach

The classification approach assumes that there are two latent classes of data, generated by either a diffusion process or a racing accumulator process. These two classes of models yield (perhaps subtly) different patterns of response times and accuracy data, such as differences in the leading edge of RT distributions or, as I reviewed above, differences in how they respond to manipulations of discriminability. A neural network can be trained to detect any differences between the classes of data, then applied to real data to classify a participant’s performance according to which model provides the closest match. The parameters of the neural network are estimated based on a variety of data simulated from both types of models, and then the trained network is applied to test data to examine how well it matches the observed behavioral data to the latent model class (Radev et al., 2021; Kvam et al., in press; Elsemüller et al., 2023). Formally, it assigns a posterior probability *p* to each model according to the input data and the training set, where the latter functions as the priors on the classification network.

To test whether a neural network could be trained to classify data generated from a diffusion or accumulator model, I ran a series of simulations where I would generate artificial data from both types of models and examine how well a deep neural network could identify which model was originally used to create each artificial data set. To do so, I simulated 100,000 artificial participants from a diffusion model and 100,000 artificial participants from an accumulator model. To create each artificial participant, I chose a random set of five parameters: drift direction $\upphi$, drift magnitude $|\mu |$, threshold $\uptheta$, non-decision time $\tau$, and drift rate variability $\nu$. This allowed us to keep the parameters consistent across relative-evidence and absolute-evidence models, matching them as closely as possible while varying only the relative directions of the options ($\upgamma = \uppi$ for diffusion, and $\upgamma = \uppi /2$ for accumulator). The model parameters for each simulated participant were drawn from the following prior distributions:

2 $$\begin{aligned} \text {Drift direction}&:&\upphi&\sim U(0,1) \end{aligned}$$

3 $$\begin{aligned} \text {Drift magnitude}&:&|\mu |&\sim \Gamma (2,2) \end{aligned}$$

4 $$\begin{aligned} \text {Threshold}&:&\uptheta&\sim \Gamma (2,2) \end{aligned}$$

5 $$\begin{aligned} \text {Non-decision time}&:&\tau&\sim \Gamma (1,.4) \end{aligned}$$

6 $$\begin{aligned} \text {Drift variability}&:&\nu&\sim \Gamma (1,1) \end{aligned}$$

Each one of these is a free parameter of the model that is estimated using the machine learning approach. Here, *U* is a uniform distribution and $\Gamma$ is a gamma distribution in shape-scale form. For the diffusion models, drift magnitude was scaled by $\sqrt{2}$ to account for the difference in the length of the projection of the accumulation trajectory onto the vector describing each option, arising from the difference in orientation relative to accumulator models (see Fig. 1).

Next, the simulated participants’ data were summarized by taking the mean accuracy; minimum (to help identify non-decision time) and mean response times for correct and incorrect responses; and five response time quantiles (.1, .3, .5, .7, and .9) for correct and incorrect responses. This resulted in a total of 15 inputs to the neural network to summarize performance from each of the 200,000 simulated participants, and a single output value corresponding to the probability that the data came from an accumulator model (outputs were coded as 0 = diffusion, 1 = accumulator).

**Fig. 4:**
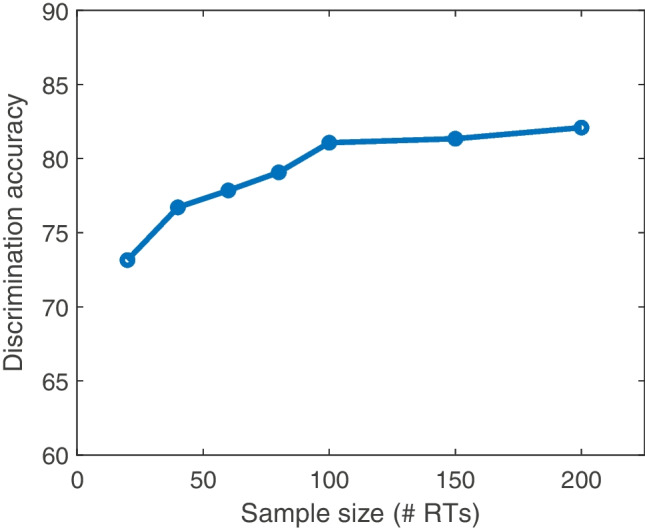
Classification accuracy of the neural network trained to discriminate between absolute-evidence and relative-evidence models (*y*) as a function of the number of simulated trials in each data set (*x*)

To examine how well the network recovered the true model as a function of the amount of data each participant provided, I repeated the same procedure while varying the samples size of each of these artificial data sets using N = 20, 40, 60, 80, 100, 150, and 200 trial experimental designs. The results are shown in Fig. 4.

In general, the network was able to classify the data correctly between 74 and 81% of the time, depending on how many trials each simulated participant completed. Classification accuracy increased with the number of trials, up to around *N* = 100. Note that this proof-of-concept uses only a single condition to discriminate between options. As I show below in the application to real data, experimental paradigms with multiple conditions and manipulations are able elicit data that make it easy to discriminate between the models. On these more realistic tasks, the classification network achieves over 98% accuracy in differentiating between relative-evidence and absolute-evidence models.

#### Estimation approach

The direct classification approach is not the only way to determine what underlying representation of evidence gives rise to observed accuracy and response times. As I reviewed in earlier sections of the paper and illustrated in Fig. 1, both relative-evidence and absolute-evidence models are special cases of a more general geometric representation of evidence (Kvam, 2019a). In this view, model identification can be carried out by estimating a free parameter, $\upgamma$, which describes the relationship between the available options in the choice set. Estimates of $\upgamma$ close to $\frac{\uppi }{2}$ would indicate an absolute-evidence, accumulator-based representation of the choice options, while estimates of $\upgamma$ close to $\uppi$ would indicate relative-evidence, diffusion-based representation.

**Fig. 5:**
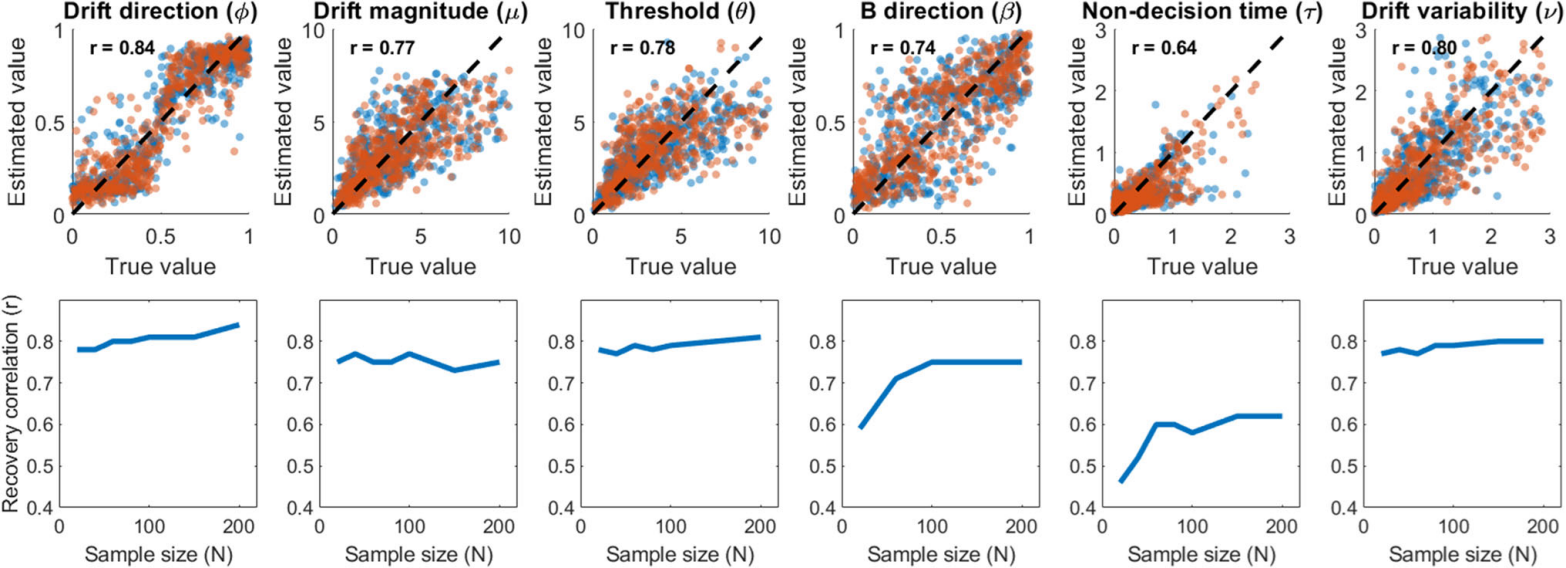
Parameter recovery for the generalized geometric accumulator model. In the *top panels*, estimation accuracy for the training set is shown in *blue* and the validation set is shown in *orange*. The *r* values correspond to the linear correlation between true and estimated parameter values from the neural network for the validation set (*orange*). The *top panels* show the parameter recovery for $N = 200$, while the *bottom panels* show how parameter recovery changes across sample sizes

Parameter estimation can be carried out using a similar neural-network based approach (Sokratous et al., 2023; Rmus et al., 2023; Kvam et al., 2023). Instead of a model indicator variable as the output of the neural network, I use the model parameters that were used to generate a particular data set. The neural network then learns the functional relationship between model parameters and data, allowing us to “invert” the simulation process and obtain the generative model parameters from the simulated data. Once trained, the network can then be applied to estimate the posterior distribution of parameters for the model it was trained on, including both the maximum a posteriori estimates and the estimated error or posterior variance (Radev et al., 2020).

To test the parameter estimation approach as an alternative to model classification, I carried out a parameter recovery study using the GSR model described above. This model adds one additional parameter $\upgamma$ to the relative-evidence and absolute-evidence models described above. As before, I generated 100,000 simulated participants from the model for each training and testing run, and tested different sample sizes from *N* = 20, 40, 60, 80, 100, 150, and 200 trials per participant. Also as before, these trials were all within a single experimental condition, limiting to an extent the performance of the network.

For each simulated participant, I used the same priors used above (Eqs. 2, 3, 4, 5 and 6) to generate simulated data. The option direction parameter was drawn from a uniform prior:

7 $$\begin{aligned} \text {Option B direction}&:&\upgamma&\sim U(0,\uppi ) \end{aligned}$$

This allowed us to explore the full range of directions that options could take relative to one another, in addition to the directions associated with relative-evidence and absolute-evidence representations.

The results of this approach are shown in Fig. 5. In general, all of the parameters of the model were recovered with reasonable accuracy. The recovery did not change too substantially across different sample sizes, and recovery of the *gamma* parameter was consistently good. It also did not change substantially between training and validation sets (RMSE for validation averaged 2.25 for the training set, and 2.39 for the validation set), indicating that there were no glaring problems with overfitting.

A greatly expanded description of the technical details of the neural networks is provided in the supplementary information.

Overall, given these results from the parameter recovery study, we should expect the estimates of the $\upgamma$ parameter – as well as other parameters of the GSR model – to be highly informative. In turn, this approach should shed light onto the structure of option representations during choice, even with extremely simple experimental paradigms featuring only a single condition. The estimation approach, as opposed to classification, has the added benefit of going beyond the simple binary comparison between absolute-evidence and relative-evidence models, and instead quantifying the relationships among options using a graded measure of option similarity. As I show below, more complex experimental designs with multiple conditions can improve further upon the network’s performance by using multiple conditions to inform the same estimates.

## Application

To examine how each of these approaches can be applied to real data, I re-analyze data from Kvam et al. (in press). In this study, participants were shown a jittering Gabor patch in the middle of the screen whose orientation on each screen update was drawn from a wrapped normal distribution (Kvam, 2019b). Their task was to determine which of two orientations displayed on the screen was closer to the average stimulus orientation. The two choice option orientations corresponded to two equiluminant lines displayed on the screen, a blue line and an orange line. Participants responded at any time by clicking the left mouse button to indicate the mean stimulus orientation was closer to the blue line, and right mouse button to indicate they thought the mean stimulus orientation was closer to the orange line. Complete details of the paradigm, as well as information about other conditions where the options changed partway through a trial (not analyzed here), are provided in the main text and supplement of Kvam et al. (in press).

**Fig. 6:**
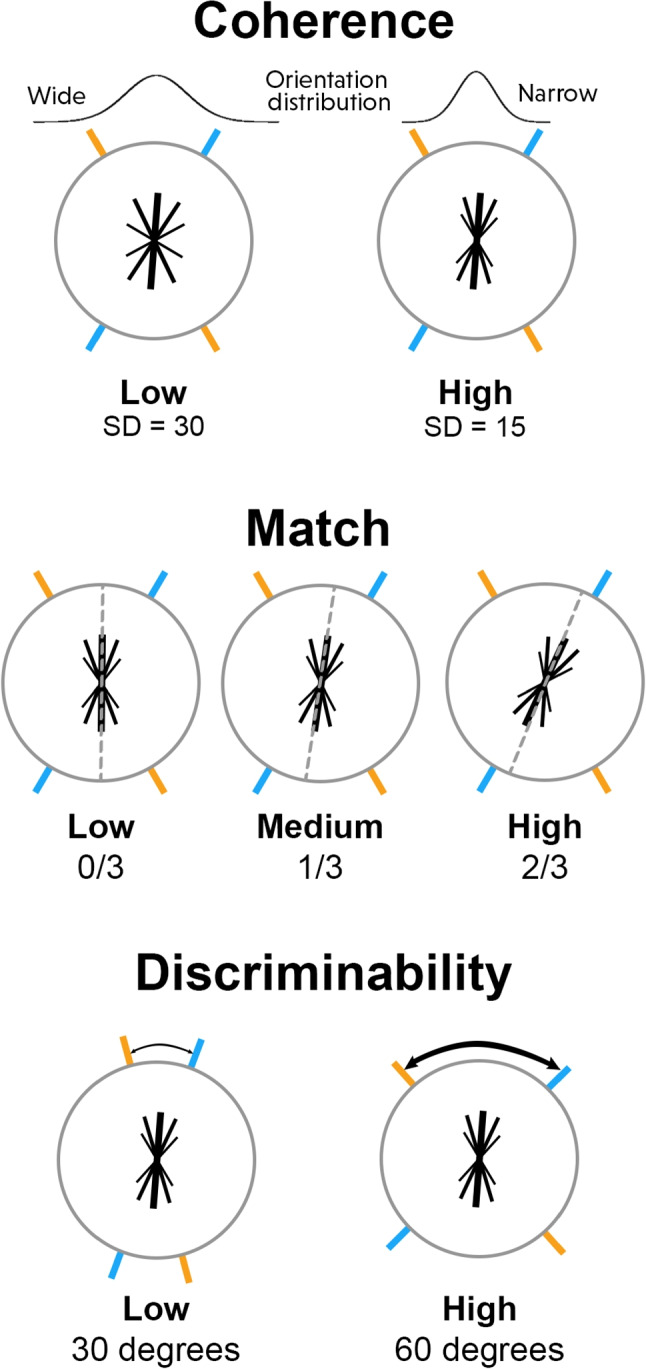
Coherence, match, and discriminability manipulations in the data set used for an application. The stimulus is displayed in the middle of the screen, symbolized by an *asterisk with a thick line* as the mean orientation and *thinner lines* as less frequently displayed orientations. The stimulus itself changes with manipulations of coherence (orientations of smaller lines corresponding to dispersion), its mean orientation changes with match, and the relative orientations of the choice options (*blue/orange lines*) change with discriminability

This paradigm allowed us to independently manipulate the coherence of the stimulus, match between stimulus and response, and the discriminability of the choice options. These are shown in Fig. 6. Coherence corresponded to the standard deviation of the wrapped normal from which the orientations of the stimulus were drawn (the stimulus noise), which could be 15 degrees or 30 degrees (two levels). Match corresponded to the orientation of the stimulus relative to the choice options (with a closer orientation corresponding to a higher match), and could be 0/3, 1/3, or 2/3 of the way from perfectly ambiguous/half between the stimuli to the average orientation perfectly matching one of the two options (three levels). A value of 0/3 meant that the mean orientation of the stimulus was exactly between the two options, while a value of 2/3 mean that the mean orientation of the stimulus was tilted 2/3 of the way toward one option over the other. Because the orientations were drawn randomly on each screen update, there was a “correct” option even on the 0/3 match trials, because the average of a random sample of orientations shown would be closer to one option or the other. Discriminability referred to how easy the two choice options should be to tell apart, corresponding to the difference in their orientations. Across trials, this was varied to be either 30 degrees or 60 degrees (two levels). The choice options were displayed for 500 ms prior to the onset of the stimulus, meaning that participants could gauge their discriminability before the start of the trial.

Each of these components – coherence, match, and discriminability – was mapped onto a different component of the evidence accumulation models. Coherence was related to the overall drift magnitude $|\mu |$, as it controlled how much information could be parsed from the stimulus on each screen update. Match was related to the drift direction $\upphi$, as it controlled how the orientation of the stimulus was related to the orientations of the choice options. Closer alignment (more similar angles between stimulus mean and choice options) resulted in a higher drift, but only by virtue of the component of the drift along the vector (*v*) describing the option being higher $\text {comp}_v(\mu )$. Although they ultimately had similar effects, separating coherence into drift magnitude and match into drift direction helped delineate these two manipulations.

Finally, the discriminability of the choice options was mapped onto the relative orientation of the choice options $\upgamma$. More similar orientations implied that the choice options were harder to tell apart – and thus had a smaller $\upgamma$ value – while less similar ones were easier to tell apart and had a larger $\upgamma$ value. Put together, these three separate parameters described the three distinct manipulations.

I reanalyzed the data from the 34 participants retained in the original data set, focusing only on trials where coherence, match, and discriminability (and nothing else) was manipulated. This resulted in 120 trials per participant, ten trials in each of the 12 conditions (two levels of coherence $\times$ three levels of match $\times$ two levels of discriminability), for a total of 4080 trials. Each trial yielded a response time and a correct or incorrect response. These data were used in each of the analyses below, demonstrating how each approach could offer insights into what model best accounted for the observed patterns of data

The data set and model code used in the current study are available on the Open Science Framework at osf.io/ctz37. This re-analysis does not constitute human subjects, but the original ethics approval was provided by the University of Florida (IRB#202100295).

**Fig. 7:**
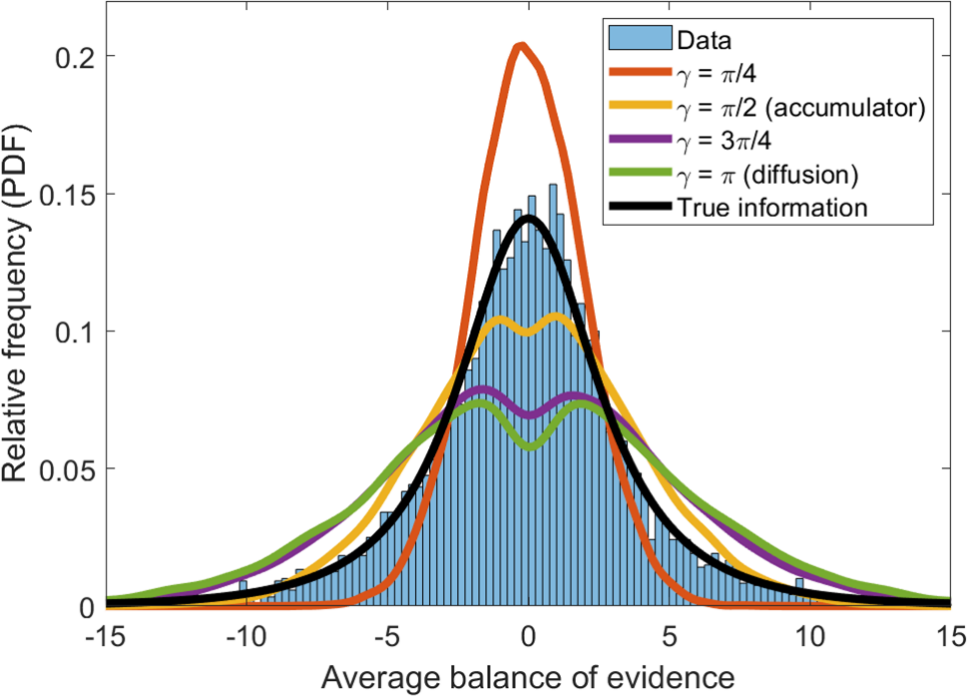
Distribution of the average balance of stimulus evidence at the end of the trial, from the real data (*histogram*), and as predicted by models with different values of $\upgamma$ (*lines*). The true information provided by the stimulus is shown in *black*, while the prediction from an accumulator model is shown in *yellow* and a uni-dimensional diffusion/random walk model is shown in *green*

### Accumulated-evidence profile

The first method for identifying generative models that I used was the accumulated-evidence profile. To form this distribution, I took the overall balance of stimulus information at the conclusion of each trial. This was calculated by finding the average orientation of the stimulus from the time it appeared to the time when a participant made a response. Orientations clockwise from the true mean orientation of the stimulus were coded as negative, while orientations counterclockwise of the true stimulus orientation were coded as positive. The sum of these coded orientations was then divided by the number of stimulus updates that took place during the trial – on average, 58.90 (SD = 34.71) frames.

The distribution of average balances of evidence across all analyzed trials of the experiment is shown in Fig. 7. As shown, the distribution of end-of-trial evidence is close to unimodal, in contrast to the bimodal distribution of support predicted by a relative-evidence model (Kvam et al., 2022). The distribution of evidence collected by participants was also fairly close to the true information provided by the stimulus, suggesting that their stopping rule, whatever it was, was not substantially distorting the relative frequencies of different levels of evidence. Generally speaking, this means the accumulated-evidence profile is likely to favor an accumulator model; however, I thought it prudent to test all models anyway to formally and quantitatively compare their predictions against the data.

To calculate the predictions of each type of model for the accumulated-evidence profiles, I simulated 100 trials from each model for each real trial, for a total of 408,000 simulated data points from each model. For each simulated trial, I drew random values for the drift magnitude, threshold, and non-decision time from the priors used in Eqs. 3, 4, and 5 and used them to create a simulated response time and response. Drift direction was permitted to vary as a function of match between the actual stimulus and the response options, while drift variability was fixed across conditions. The option B direction was fixed for each model: $\uppi /2$ for the accumulator model and $\uppi$ for the diffusion model.

This approach allowed us to match the structure of the real data while creating a large simulated data set with a variety of combinations of parameter settings, reflecting the variability across participants, conditions, stimuli, and trials. In total, I generated predictions from eight models with values of $\upgamma$ ranging evenly from $\uppi /8$ to $\uppi$.

To test for bimodality, I took two approaches. The first approach involved comparing the observed distribution of information to an unbiased sample. To do so, I calculated the K-L divergence between the predicted distribution from the model and the observed distribution. Using cubic spline-based interpolation to estimate the best-fitting value for the aggregate distribution from the eight different models, I found that the value of $\upgamma$ was approximately 1.22, or $.39 \uppi$. This puts it closest to an accumulator model, although the angle between options is actually slightly narrower.

Second, I calculated Sarle’s bimodality coefficient (Freeman & Dale, 2013; Pfister et al., 2013; Institute, 1990), which seeks to quantify the degree of bimodality in a sample distribution. The coefficient *B* is calculated as

8 $$\begin{aligned} B = \frac{g^2 + 1}{k + \frac{3(n-1)^2}{(n-2)(n-3)}}. \end{aligned}$$

where *g* is an estimate of the sample skewness, *k* is an estimate of the sample kurtosis, and *n* is the number of data points in the sample.

This coefficient *B* varies from 0 to 1, where 0 is a perfectly unimodal distribution and 1 corresponds to a distribution that takes on exactly two separate values. The calculated bimodality coefficient for several example distributions, including relative-evidence and absolute-evidence models, is shown in Fig. 2. As we might expect, it is generally quite close to zero, but gets as high as $B =.25$ for a relative-evidence model. For the present sample, the bimodality coefficient was $B = 0.08$, indicating relative low bimodality consistent with absolute-evidence models.

For the K-L divergence, there was not enough individual-level data to adequately assess the accumulated-evidence profiles for each participant. This was partly due to the fact that the trials used to evaluate the models here constitute only a quarter of the data that was collected – the majority of which focused on how people made decisions among options that changed over time (Kvam et al., in press). It may take several sessions of data – on the order of 1000 trials – to obtain distributions of stimulus evidence that are sufficient to identify conclusively which generative model shown in Fig. 7 provides the best account of the data. Here, we can do so on the aggregate level, allowing us to make inferences about the overall tendency across participants to follow an absolute or relative stopping rule.

For the bimodality coefficient, we could actually compute *B* for every participant. This gives us some insight into the shape of individual-level accumulated-evidence profiles. The average *B* when calculated on the individual level was somewhat higher than the aggregate value, at $M(B) = 0.15$ ($SD = 0.03$). This is closer to halfway between absolute-evidence and relative-evidence models, indicating that individual participants may not so cleanly categorized into one or the other on the basis of bimodality alone. Of course, bimodality does not capture the whole picture – as other features like the variance of the distributions are useful for categorizing them. the bimodality coefficient is also limited somewhat in that it does not have an associated probability density function, making it difficult to capture uncertainty about the aggregate or individual-level bimodality.

As I suggested when introducing this method, accumulated-evidence profiles can start to break down when there is substantial non-decision time during a trial, during which the stimulus might update even though the participant is not considering new information. This can dilute the final distribution of evidence by adding a true random sample to the biased sample resulting from a particular stopping rule. Therefore, while this result points us in the direction of an accumulator model, it is not conclusive for this type of experiment where the stimulus information rate is quite high (60 Hz).

### Effect of discriminability

The second method for evaluating whether a set of data is better fit by an accumulator model or diffusion model is to examine how accuracy and response times change in response to a manipulation of the discriminability of the two options. As described above, the experiment included two levels of discriminability, where the options were 15 degrees or 30 degrees away from one another.

**Fig. 8:**
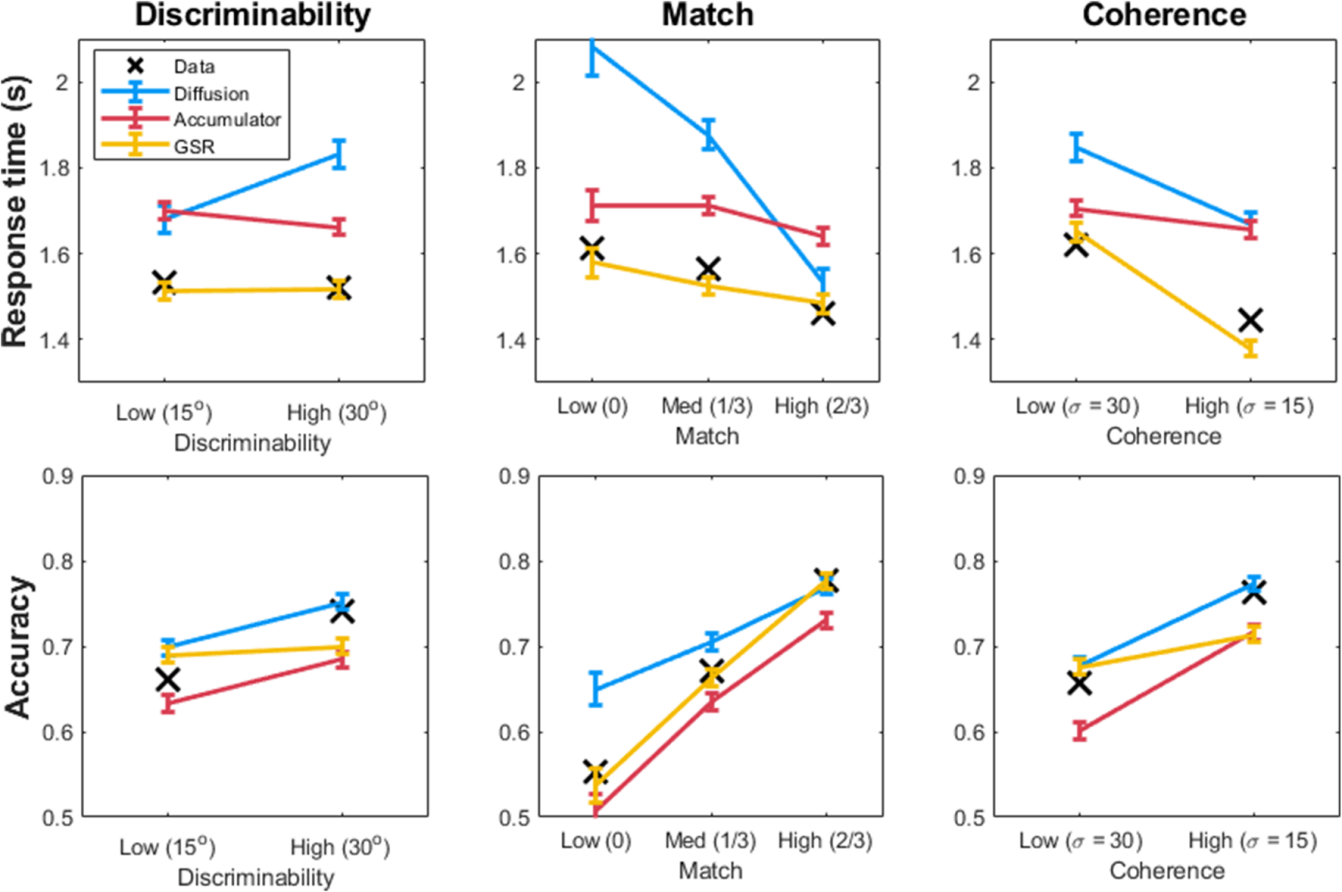
Posterior predictions from the diffusion model (*blue*), accumulator model (*red*), and GSR model (*yellow*) for the patterns of mean response times (*top*) and accuracy (*bottom*), plotted against the data (*black x*). *Error bars* correspond to the predicted standard error of the mean from a single simulated data set generated by the best-fit parameters of each model

In the experimental results, there was no change in mean response times between the low-discriminability (M = 1.52s, 95% HDI = [1.49, 1.55]) and the high-discriminability condition (M = 1.52s, 95% HDI = [1.50, 1.57]), and a Bayes factor analysis favored the null / no effect of this manipulation on RT (BF$_0$ = 25). However, there was strong support (BF > 100) for a modest increase in choice accuracy from the low-discriminability (M = .66, 95% HDI = [.64, .68]) and the high-discriminability condition (M = .74, 95% HDI = [.72, .76]). This result, as well as the effects of coherence and match on response time and accuracy, are shown in Fig. 8.

The diffusion model had particular difficulty accounting for the effect of discriminability, as it tended to predict either an increase in response time alongside an increase in accuracy, corresponding to an increase in threshold from the low- to the high-discriminability conditions. It had difficulty handling cases where one increased or decreased without the other, or where there was clearly a difference in drift creating faster response times alongside higher accuracy in high-discriminability conditions.

By contrast, the accumulator model had no such trouble with the pattern of response time and accuracy data across low- and high-discriminability conditions. As shown in red in Fig. 8, it predicted little difference in mean response times and an increase in accuracy with greater discriminability, corresponding well to the data. The reason for this is that an increase in drift rate or support for one option, and in particular the disfavored option, can decrease accuracy while leaving response times largely unaffected. It is not constrained in the same way as the diffusion model to predict strong covariance between accuracy and response time.

As with accumulated-evidence profiles, the discriminability-manipulation approach is not entirely conclusive on its own, but it certainly hints that there are patterns in the data that cannot be accounted for by a traditional diffusion model, but can be accounted for by an accumulator-based model. In addition to the accumulated-evidence profiles, it lends a second source of support for an absolute-evidence stopping rule and an accumulator model of this data set.

### Model classification network

The most straightforward approach for model comparison using machine learning is the model classification network, which takes a set of data and identifies the most likely generative model based on a set of inputs, such as accuracy and response time distributions. For this set of data, there were 12 different conditions: two levels of discriminability, two levels of coherence, and three levels of stimulus-option match. This provided a great deal more information to discriminate between models than the single-condition demonstration above.

To train the classification network, I generated 500,000 simulated data sets from the accumulator ($\upgamma = \uppi /2$) and diffusion ($\upgamma = \uppi$) models, using the same priors specified in Eqs. 2-6. Each simulated data set perfectly replicated the experimental procedure of one of the experiment participants in terms of the condition and trial structure, ensuring that they corresponded to the paradigm used to elicit the real data as closely as possible. This allowed the classifier network to match as closely as possible the real data to which it was applied.

One thing to note about these models is that the diffusion models have one drift rate, while the accumulator models inherently have two (one for each accumulator). However, the two drift rates in the accumulator model are ultimately redundant with other parameters, especially when realized in the GSR framework. To account for this, the drift rates in the accumulator model were set by first evaluating the proportion of samples that would favor the correct or incorrect option, then multiplying this value by the drift rate (a free parameter for each condition), and finally adding the drift rate variability to each accumulator. This allowed us to use a single parameter to specify both drift rates in the accumulator model. The exact details are provided in the supplementary material. Critically, this allowed us to match the number of parameters between the relative-evidence and absolute-evidence models, ensuring that differences in model complexity – at least as far as the number of parameters is concerned – was not responsible for these results.

For each simulated data set, I summarized the observed data in terms of 96 inputs: the mean accuracy, mean response time for corrects and incorrects, and five response time quantiles (.1, .3, .5, .7, and .9) for each of the twelve conditions. These inputs were matched with an indicator variable that served as the output of the neural network, which was 0 for data simulated from the diffusion model and 1 for data simulated from the accumulator model.

**Table 1:** Confusion matrix illustrating the proportion of times that the trained neural network was able to classify the observed data according to its generative model

		Recovered model	
		Diffusion	Accumulator
True model	Diffusion	98.36%	1.64%
	Accumulator	1.48%	98.52%

Rows correspond to the model used to create data, while columns correspond to the model that was inferred by the network

Once the network was trained on the data simulated from each model, I tested its performance on a hold-out set of data to ensure its accuracy on out-of-sample data. This is referred to as the *validation set*. The confusion matrix for the trained network is shown in Table 1. Overall, the network was able to correctly classify (assign a posterior likelihood greater than 50%) the correct model from the data over 98.4% of the time.

Next, the model was applied to the real data. To format the data for input to the network, I concatenated the mean response time, mean accuracy, and response time quantiles for each condition for each participant. This yielded a set of 96 inputs for each person who took part in the experiment. These inputs were then fed into the trained network to yield a posterior probability of the model (diffusion or accumulator) given the data.

**Fig. 9:**
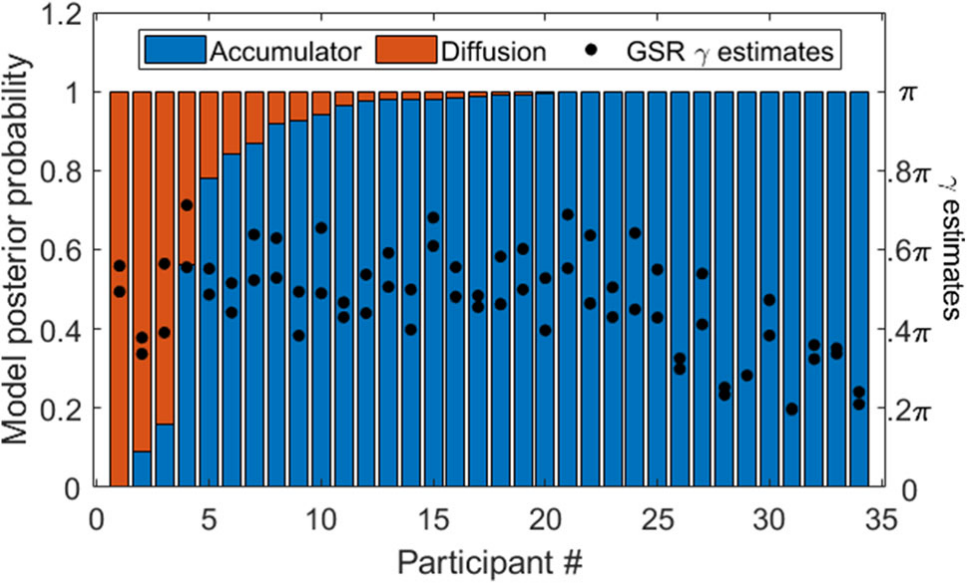
Posterior probabilities assigned to accumulator (*blue*) and diffusion (*red*) models by the model comparison network for each participant, organized from most strongly favoring diffusion/relative evidence to most strongly favoring an accumulator/absolute evidence representation. Also shown are the estimates of the $\upgamma$ parameter from the GSR models: higher values tend to favor a relative evidence/diffusion representation, while lower values tend to favor an absolute evidence/accumulator representation

The resulting posterior probabilities are shown in Fig. 9. Out of 34 participants, only three were best fit by a diffusion model (overall posterior probability = .11), while the remaining 31 were better fit by an accumulator model (overall posterior probability = .89). This lends strong support to the claim that participants were using an accumulator- as opposed to a diffusion-based representation of their choice options.

### Parameter estimation network

Understanding the representation of evidence during decision-making entails more than just a comparison between relative-evidence and absolute-evidence models. These are only two out of a continuum of possible ways that participants might be representing their options during choice. For a deeper understanding of how participants are representing their options, it is informative to estimate $\upgamma$ as a free parameter using an approach like the GSR rather than comparing between two particular values.

To estimate the more general model, I trained a second neural network to estimate the parameters of a full GSR model of performance on the task. This model consisted of a total of 14 parameters. It included six of the same parameters as the proof-of-concept demonstration shown in Fig. 5: drift direction, drift magnitude, threshold, non-decision time, choice option direction, and drift variability parameters. It also added start point variability and allowed drift direction to vary with the match manipulation (three levels), the threshold and option directions to vary with the discriminability of the choice options (two levels each), and the drift magnitude to vary with the coherence of the stimulus (two levels) and level of match (two levels; mean drift was set to 0 in the lowest-match condition because the stimulus did not favor either option on average). Drift variability, start point variability, and non-decision time were fixed across conditions.

These modeling choices were principled choices based on hypotheses about what components of the model change with different manipulations, following from previous work connecting different parts of the GSR to stimulus manipulations (Kvam, 2019a; Kvam et al., in press, 2023). It is certainly possible to test more or less complex versions of the model and compare them for best fits, but the goal here is not model comparison so much as using a good model to estimate the $\upgamma$ parameter.

**Fig. 10:**
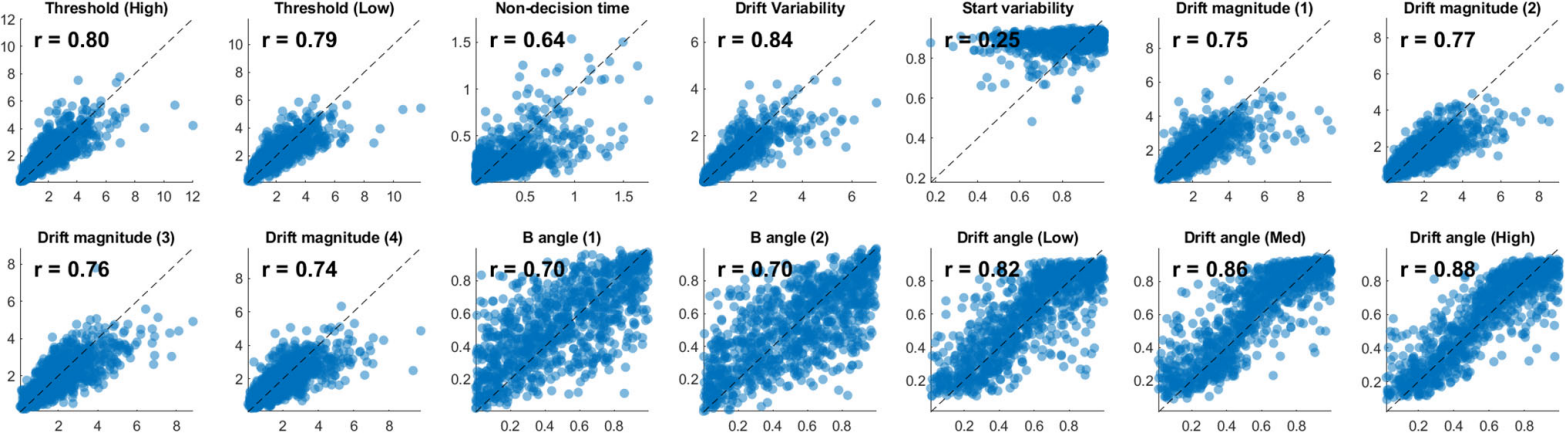
Parameter recovery for the GSR model on the full data set. Each subplot displays a scatterplot of 1000 true (*x*)- versus estimated (*y*)-values of a different parameter of the GSR. To ensure that all resulting parameter values are positive, all output parameters given to the network were log-transformed for training and validation. Thresholds and alternative angles ($\upgamma$) were permitted to vary with discriminability, drift magnitudes vary with the degree of stimulus coherence, and drift directions vary with the degree of match between stimulus and choice alternatives. This allows the parameters to be estimated with greater precision than the single-condition estimates shown above in Fig. 5

In general, the network was able to recover the parameters of the model well, as shown in Fig. 10. Its performance was generally much better than that of the initial demonstration (Fig. 5, due to the larger number of conditions.

The parameter estimates of $\upgamma$ are shown in Fig. 9 as a pair of dots overlaid on the bars. The y-axis on the right indicates the values of $\upgamma$ for the two discriminability conditions for each participant. If the two approaches converge, we should see the probability of the diffusion model decrease alongside the estimates of $\upgamma$, i.e., the height of the red bar should covary with the height of the dots (and the height of the blue bars should inversely covary with the height of the dots).

This is exactly what happened. On average, the estimates of $\upgamma$ for the high-discriminability condition were .51$\uppi$ (SD = .14$\uppi$) choice options evidence corresponding to an accumulator model. In addition, the estimates of $\upgamma$ tended to decrease as the probability of an accumulator model increased. The model posterior probability and $\upgamma$ estimates for each person were correlated, with Spearman’s $\uprho$ = -.56 for high-discriminability and $\uprho$ = -.49 for low-discriminability $\upgamma$ estimates.^4^ This provides converging evidence between the model classification and parameter estimation approaches, which tended to agree upon which participants were exhibiting behavior corresponding to an accumulator-like representation of the choice options and which participants were exhibiting behavior closer to that of a diffusion model.

In addition to the GSR model, I also fit relative-evidence and absolute-evidence models of performance in order to assess their performance in accounting for the data overall. These models used the same baseline parameters of drift, threshold, non-decision time, start point variability, and drift rate variability with the value of option direction $\upgamma$ set to either $\uppi$ (diffusion) or $\uppi /2$ (accumulator). For these two models, the drift rates were permitted to vary across discriminability, match, and coherence measures, in line with traditional approaches to modeling these manipulations (Ratcliff et al., 2016; Heathcote & Matzke, 2022). Threshold was additionally permitted to vary with the discriminability manipulation, as the choice options (and thus their discriminability) were known before stimulus onset in the experiment. The parameter recovery and additional details for these two models are provided in the supplement.

The resulting performance of the GSR (less complex) and absolute-evidence and relative-evidence (more complex) models are shown in Fig. 8. As shown, the GSR generally accounted well for the patterns of accuracy and response time across conditions, and tended to do so better than both absolute-evidence and relative-evidence models despite these models allowing drift direction and magnitude to vary across more conditions. These results suggest that the GSR is not only a method for identifying the representation of choice options in experiments, but a viable model of 2AFC tasks in its own right.

One final issue for the parameter estimation networks is to quantify uncertainty using something akin to a confidence interval or Bayesian posterior. Previous work has explored approaches to estimating posterior variance (Radev et al., 2020; Sokratous et al., 2023), and more recent work has shown that adding a dropout layer to the neural network can actually allow efficient sampling from the posterior of a Gaussian process relating the data to model parameters (Gal & Ghahramani, 2016; Kvam et al., 2024). Alternative approaches using Bayesian neural networks (Izmailov et al., 2021) may yield even better characterizations of posterior uncertainty when the assumptions of a Gaussian process are violated.

## Discussion

Here, I identified four methods for contrasting the stopping rules implemented by different types of models. I focused specifically on a diffusion model implementing a relative-evidence stopping rule, and an accumulator model implementing an absolute-evidence stopping rule. While not exhaustive in terms of the models that are used in the choice literature, they are certainly representative of the two biggest classes of models used in these domains. Moreover, these types of models are special cases of a more general spatial or geometric representation of evidence – two points along a continuum of interactions between support between available options. The results indicate that there are multiple ways to tell apart such models: (1) by looking at the information a decision-maker considered en route to their choice, (2) by looking at specific manipulations of discriminability and how the impact accuracy and response time, (3) by using machine learning to classify observed data by generative model, and (4) by directly estimating the relationships between choice options in the geometric framework.

Each of these approaches can shed light on the representations of evidence involved in decision-making, as I illustrated with the application to data from a binary choice task. Ultimately, the four approaches converged on a common conclusion: an accumulator / absolute-evidence representation provided a better account of the data than a diffusion / relative-evidence representation. Those methods that could go beyond the binary inference – the approach based on accumulated-evidence profiles (one above) and the parameter-estimation approach (four above) – took it a step further and hinted that the representation of choice options likely involved slight mutual excitation.

It is critical to note that these conclusions are specific to the experimental data under consideration. The task, where participants decided between a pair of orientations who were always within 60 degrees ($\uppi /3$) of one another, might naturally lend itself to an accumulator-based representation of evidence. The goal of this application was not to demonstrate that one model is universally better than another, but to show that these four methods can all shed light on the representations of evidence underlying two-alternative choice. The convergence between the four methods was unexpectedly high, indicating that each one is likely arriving at a reliable conclusion. I expect, or at least hope, that applications of these approaches to other data sets will result in similarly strong conclusions.

### Use requirements and recommendations

The different approaches presented here range in terms of how widely and easily they can be applied. Accumulated-evidence profiles are arguably the most difficult to use, because they require precise stimulus timing and a record of everything that a participant saw on a given trial in order to identify the evidence that they might have accumulated. Using the bimodality coefficient, it appears applicable as long as a few hundred decisions are collected from each participant; the relatively low variability in estimates of *B* across participants suggests that it should work for typical cognitive experiments. The K-L divergence requires significantly more data, as it can be highly sensitive to outliers and involves matching the entire observed distribution to an expected one. It is best used when there is tight stimulus control and ideally discrete sampling, although accumulated-evidence profiles still suggest bimodality even in the continuous case. Overall, accumulated-evidence profiles are best used when the stimulus information arrives relatively slowly (minimizing the impact of non-decision time) and the information presented to participants can be precisely tracked.

The disentangling approach is slightly more flexible, in that it can be used whenever there is an effective manipulation of option discriminability relative to true stimulus information. It relies on there being stimuli that are ambivalent and options that are highly discriminable – which results in longer response times the higher the value of $\upgamma$. Therefore, it can be used as long as option discriminability is manipulated separately from stimulus-option match.

Finally, the machine learning approaches are by some distance the most widely usable approaches to discriminating between different models. Existing work on using neural networks to classify data into generate models have suggested that this approach has high accuracy and significantly out-performs model comparison metrics like BIC, AIC, WAIC, squared error, and log likelihood (Elsemüller et al., 2024; Kvam et al., 2024, in press; Radev et al., 2021). It should therefore be able to classify the generative model for a set of accuracy and response time data with high fidelity.

Likewise, the parameter estimation approach has been shown to be accurate even with very sparse data (as few as 20 data points) (Sokratous et al., 2023; Radev et al., 2020). The main barrier to using the geometric approach I presented here has been a practical one – namely, that analytical likelihoods do not exist except for specific cases like the circular diffusion model (Smith, 2016; Kvam & Turner, 2021). However, amortized Bayesian methods like neural networks circumvent the need for analytical likelihoods – meaning that the more general model with the $\upgamma$ parameter can be widely used. Indeed, there should be no special requirements to apply the parameter-estimation and model-comparison methods where machine learning is used and it is likely they can be applied to many existing data sets as well.

### Limitations and future directions

This paper focused exclusively on two-alternative choice, which has been the traditional domain of application for both relative-evidence and absolute-evidence models. While many choice paradigms use this structure, 2AFC tasks are by no means the only paradigm researchers use to study choice. These competing representations of the decision process should also be compared in the multi-alternative choice case. Early investigations into these differences have in some cases already been carried out (Turner et al., 2018; Evans et al., 2019), although they have not tested the GSR approach to modeling. The relationships between choice options become particularly important in multi-alternative choice because these interactions are thought to drive context effects (Trueblood, 2022), which are key phenomena to accommodate if we seek to create more universal models of decision-making.

The machine learning approach I used here for model fitting and comparison is particularly well suited to models lacking an analytic likelihood (Sokratous et al., 2023), although models with these likelihood functions allow us to check their performance against an objective standard (Radev et al., 2020). The GSR model with a free $\upgamma$ parameter would be significantly more difficult to fit without the use of neural networks, with other approaches like probability density approximation (Turner & Sederberg, 2014; Holmes, 2015) being computationally costlier and slower. While model comparisons using neural networks tend to compare favorably against likelihood-based methods of model comparison (Radev et al., 2021), the lack of likelihoods here means there is no basis for comparison. Of course, requiring likelihoods would mean we could not entertain many of the models used here anyway – meaning we would lack the flexibility to examine the many interesting hypotheses related to the GSR.

These are not the only ways to tell the differences among models. More traditional methods for model comparison and other types of stimulus manipulations have sometimes been able to discriminate between absolute-evidence and relative-evidence models (Heathcote & Hayes, 2012; Donkin et al., 2011). Early work by Busemeyer (1985) was also able to show that the last piece of information a decision-maker gathers is highly informative as to what stopping rule they are using, which should also be informative for discrete-step random walk and accumulator models. Other effects like magnitude sensitivity (Pirrone et al., 2022) hint at accumulator representations, but are not conclusive in the sense that they do not rule out diffusion models where noise increases with stimulus magnitude (Ratcliff et al., 2016).

I deliberately limited the scope of the investigation to relative-evidence and absolute-evidence stopping rules for the same underlying distributions of stimulus evidence so that the endeavor would be tractable, and so that I could directly compare models that were well matched. There are many other models that one might consider for two-alternative forced-choice paradigms, such as a linear ballistic accumulator (Brown & Heathcote, 2008) or leaky competing accumulator model (Usher & McClelland, 2001). Short explorations of these two models are provided in the supplement, as is a comparison between all five modeling approaches (diffusion, accumulator, GSR, LBA, and LCA) for this data set. There are many other models we could entertain, and certainly future work expanding on these results should do so, but the comparison of absolute-evidence and relative-evidence models provides a proof that these methods are capable of discriminating among different models of choice and response time.

More generally, we might explore structural differences in evidence representations for different models rather than specific models of interest. Additional mechanisms like ballistic accumulation (Brown & Heathcote, 2005), evidence decay (Heath, 2000), collapsing choice boundaries (Hawkins et al., 2015), distributed evidence representations (Zandbelt et al., 2014) and interactions between accumulators (Usher & McClelland, 2001) are all questions that might answered by looking at specific types of manipulations, patterns of information search, or using new tools in machine learning for model comparison and parameter estimation among models that were previously intractable. Some of these are examined in the supplement, including a linear ballistic accumulator model and a model with evidence decay and lateral inhibition.

One component that has recently grown in popularity is the idea that responses may be driven by a dynamic signal, either due to collapsing choice boundaries or due to an urgency signal (Smith & Ratcliff, 2022; Hawkins et al., 2015; Hawkins & Heathcote, 2021; Palestro et al., 2018; Evans et al., 2020; Zhang et al., 2014). Part of the difficulty of testing these models is that they make it difficult to derive an analytic likelihood. Likewise, multi-stage models of decision-making can be difficult to fit (Diederich & Oswald, 2016; Diederich & Trueblood, 2018) due to the stochastic or latent attentional processes that drive switching between considering different attributes. This makes machine learning or amortized estimation (Radev et al., 2021, 2020; Sokratous et al., 2023) used here an attractive option for model fitting and comparison. However, evidence for these types of models has been mixed (Voskuilen et al., 2016; Smith & Ratcliff, 2022) and the exact method by which urgency is implemented is not yet clear.

The use of a two-dimensional space as in the geometric approach further complicates and illustrates the potential complexity of collapsing boundaries. In a uni-dimensional representation, collapsing boundaries are relatively simple – a modeler simply needs to specify a hazard function that describes how and when the boundaries get lower. However, the collapse of the boundary becomes a multivariate problem when we move to two dimensions. Instead of relating boundary height to time, we must instead map the shape of the boundary location across two (or more) dimensions to time. If each option is associated with a single choice boundary, or if the whole accumulation process unfolds in a hypersphere (Smith, 2016; Smith & Corbett, 2019), it might be possible to use a similar approach to the univariate case – reducing the height of all boundaries according to the same hazard function. Yet it is theoretically possible to have different boundaries that reduce at different rates, boundaries that do not form convenient shapes (Kvam et al., 2023), some boundaries that do not collapse while others do, or even boundaries that are controlled by a secondary timing process (Hawkins & Heathcote, 2021). While the present results do not encapsulate these models, the methods I present for analyzing and discriminating among the different possibilities can greatly enhance the model fitting and comparison process for collapsing-boundary models.

### Conclusions

Although a clear delineation between absolute-evidence and relative-evidence models by itself is useful, I hope that what this paper delivers is in many ways more useful. This included a general framework for understanding how evidence relates to stopping rules in decision models, four approaches to analyzing data that identify underlying representations of the choice options, and a demonstration that they converge on the same conclusions. By applying this approach to many data sets, modelers may begin to unravel the diversity of stopping rules that people use to make their decisions and identify how differences in tasks relate to differences in representations of evidence and choice options. I hope that this allows modelers to explore new and more complete models, deepening our understanding of the latent processes governing decision-making.

## Open Practices Statement

The data, analysis code, and models are available at https://osf.io/ctz37/. They were not preregistered.

## Supplementary Information

Below is the link to the electronic supplementary material.

Supplementary file 1 (pdf 837 KB)

## Appendix A: Mathematical model specification

The main text focuses on providing an accessible overview of the models, but this can occasionally come at the expense of precision as it relates to things like the mathematical specification of the models. To help readers familiar with these models and their notations to follow the logic, I present a formal specification here.

Both the relative-evidence and absolute-evidence models assume that a person’s preferences or beliefs at time *t* are represented in the state *s*(*t*). This state can be multidimensional, but here I focus on a two-dimensional representation, which is sufficient to describe most existing models. As a decision-maker considers new information, their state changes over time. In the discrete-time framing used for the accumulated-evidence profiles, this state changes as

9 $$\begin{aligned} s(t+1) = s(t) + N(\mathbf {\mu }, \Sigma ) \end{aligned}$$

Here, $N(\mathbf {\mu }, \Sigma )$ is a bivariate normal distribution. Its average movement follows the vector $\mathbf {\mu }$, whose two dimensions describe the movement in the *x*-direction and *y*-direction. Variability in the accumulation process is described by the covariance matrix $\Sigma$. For these present purposes and most applications of multidimensional diffusion decision models, the dimensions are uncorrelated (Smith, 2016, 2019), although correlations across dimensions can be used to specify spatial dependencies (Kvam & Turner, 2021; Ratcliff, 2018). For now, I assume no correlations and assume equal noise along each dimension, meaning that

10 $$\begin{aligned} \Sigma = \begin{bmatrix} \upsigma ^2 & 0 \\ 0 & \upsigma ^2 \end{bmatrix} \end{aligned}$$

It can therefore be specified with a single parameter – the diffusion rate $\upsigma$. This makes each sample $N(\mathbf {\mu }, \Sigma )$ radially symmetric, which becomes important when we start to compare relative-evidence against absolute-evidence models.

As each step in the accumulation process and the time between each step gets very small, this process converges to a two-dimensional diffusion process specified by the differential equation

11 $$\begin{aligned} ds = \mathbf {\mu } dt + \Sigma dW(t) \end{aligned}$$

where *W*(*t*) is a two-dimensional Brownian motion process (Smith, 2016).

In the geometric framework, the degree of support for a given option is given by taking the state *s* and projecting it onto a vector *v* corresponding to one of the choice options. For example, if we have choice option A specified by vector $v_A = [1,0]$, then the degree of support for option A $s_A$ at a given time *t* is given by the component of the state along the alternative vector:

12 $$\begin{aligned} s_A(t) =\text {comp}_{s_A}(s(t)). \end{aligned}$$

In this case, the degree of support for option A will simply be the x-coordinate of the current state. Here is where radial symmetry becomes important. Because the change in any direction *v* is normally distributed, the change in support for every option under consideration will likewise be normally distributed. Specifically, the drift rate for any option $v_j$ will be $\text {comp}_{v_j}(\mathbf {\mu })$ and the diffusion rate will simply be $\upsigma ^2$.

This accumulation process is common to all the models presented in the main text. The only difference among them is how the alternatives are oriented relative to one another. To form a diffusion decision model, a relative-evidence boundary must be set such that the two options are oriented in opposing directions, $v_A = -v_B$, or rather $\text {comp}_{v_A}(v_B) = -1$. To form a racing diffusion model where support for the two options is independent, an absolute-evidence boundary must be set such that the vectors specifying the two options are orthogonal, $\text {comp}_{v_A}(v_B) = 0$. The geometric model all other orientations of the alternatives, spanning $-1 \le \text {comp}_{v_A}(v_B) \le 1$.

For each of these models, response time is determined by two parts: the number of steps or amount of time it takes the process to cross one of the boundaries, and the non-decision time. Non-decision time is typically specified as a free parameter in both absolute-evidence and relative-evidence models, although estimates may diverge depending on which approach is used (Donkin et al., 2011; Heathcote & Hayes, 2012). Non-decision time can easily be matched across models, either as a single value or as a (typically, uniform) distribution (Ratcliff & McKoon, 2008):

13 $$\begin{aligned} RT = \Gamma (n,t) + \tau . \end{aligned}$$

Here, *n* is the number of steps in a simulated evidence accumulation trajectory before reaching a decision and *t* is the number of steps, commonly set at 10 ms for a close approximation of a continuous process. Technically, a continuous, memoryless (i.e., Markov) random walk should use a gamma distribution to map the number of steps to the response time because the time *t* between each step must be exponentially distributed (Ross et al., 1996), making the sum of *n* steps gamma-distributed. However, many modelers will simplify this by assigning a fixed value to each time step, making the response time equal to simple $n \cdot t + \tau$.

## Footnotes

1 Technically, both relative-evidence and absolute-evidence models can include a diffusion process (e.g., Tillman et al., 2020), although colloquially “diffusion” models refer to the diffusion decision model originating with Ratcliff (1978). For this reason, I try to focus on relative evidence and absolute evidence as the defining names of each model class – but occasionally refer to relative-evidence models as “diffusion” or absolute-evidence as “accumulator” models.

2 It is also possible to have “negative” choice boundaries for rejecting option A or B, which would be situated opposite options A or B (Kvam, 2019a; Diederich & Mallahi-Karai, 2023). However, typical accumulator models lack this component, so it is not included in the models I analyze here.

3 I use degrees here to keep things simple during the introductory description of the model. When wading into technical details and fitting this model, the angles that $\upgamma$ can take are presented in units of radians rather than degrees.

4 I use a rank-based correlation coefficient because while the relationship between the two should be decreasing, there is no reason to expect that it will be linear.
