# Supplementary material for: The Tweedledum and Tweedledee of dynamic decisions: Discriminating between diffusion decision and accumulator models
Source: Psychon Bull Rev. 2024 Oct 1;32(2):588–613. doi: 10.3758/s13423-024-02587-0 (PMC12000211; doi:10.3758/s13423-024-02587-0)
Supplement: Supplementary file 1 — (pdf 837 KB) [file 13423_2024_2587_MOESM1_ESM.pdf]

Supplementary materials for "The Tweedledum and Tweedledee of dynamic decisions: Discriminating between diffusion and accumulator models"

Peter D. Kvam
Affiliation

In the main text, we explored three main models of the decision process: (1) a unidimensional random walk / diffusion decision model using a "relative" stopping rule where a decision is triggered when the balance of support between two options tips far enough in one direction; (2) an accumulator model using an "absolute" stopping rule where a decision is triggered when one option gathers enough support to be chosen; and (3) a general framework for representing evidence accumulation during decision making that encompassed both the diffusion and accumulator models we tested. However, these only span a few of the most common models used in dynamic decision-making. All of the models we tested had within-trial noise (normally distributed), but did not include mechanisms like leakage or lateral inhibition that are commonly used to capture how support for different options changes over time (Turner et al., 2018; Trueblood et al., 2014; Trueblood, 2022; Usher & McClelland, 2001, 2004; Busemeyer et al., 2019).

In these supplementary materials, we explore two additional models of binary choice – the leaky competing accumulator model and the linear ballistic accumulator model. We also provide the parameter recovery details and parameter estimates for the models that were fit in the main text.

Additional model fitting details

Each of the models we fit in the main text were trained and tested using a deep neural network-based approach based on the work of Sokratous et al. (2023). In these networks, the inputs were observed data, summarized in terms of the accuracy, minimum and mean response time for correct and incorrect responses, and response time quantiles for each condition. The outputs were the generative model parameters. In the case of the real application, we used diffusion and accumulator models with 17 total parameters: two thresholds, permitted to vary with option discriminability; one non-decision time; one start point variability parameter; one drift rate variability parameter; and twelve drift rates, one for each condition. For the accumulator models, the drift rate for the second (incorrect) option was drawn as one minus the drift rate for the correct option, which was bounded between 0 and 1.

The network itself had four hidden layers, and the number of nodes in each hidden layer got progressively smaller. The input layer had 84 inputs (12 conditions $\times$ 7 condition summary statistics), which fed into a fully-connected hidden layer with 60 nodes. The next hidden layers had 40, 25, and then 17 nodes, allowing it to successively compress the information in the inputs down to the number of outputs. Each hidden layer had a hyperbolic tangent activation function (tanh),

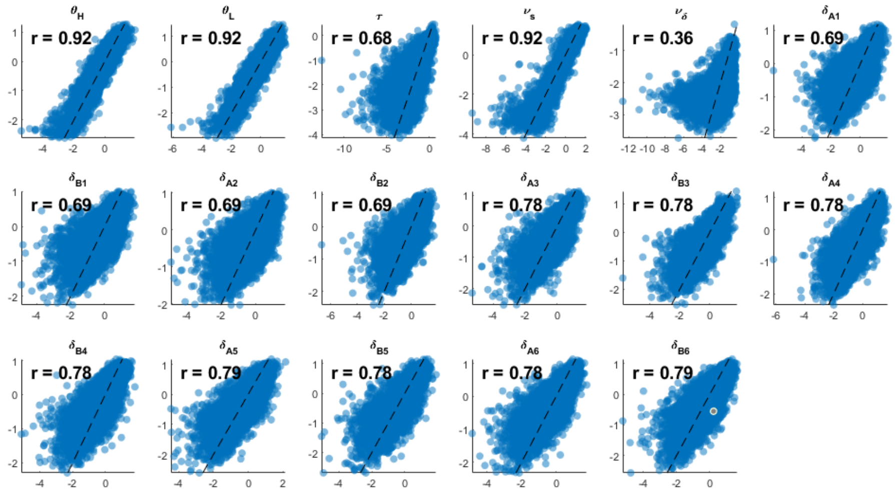

Figure S1

Parameter recovery study for the diffusion model. Each subplot displays a scatterplot of 1000 true (x) versus estimated (y) values of a different parameter of a diffusion model with relative-evidence stopping rule. To ensure that all values are positive, all parameters have been log-transformed for estimation. The parameter θ *corresponds to thresholds (2),* τ *to non-decision time,* ν_s *to start point variability,* ν_δ *to drift rate variability, and* δ *parameters correspond to the drift rates for each of the twelve conditions in the study.*

which has tended to work better that other activation functions like ReLU, leaky ReLU, or logistic functions (Sokratous et al., 2023).

As stated in the main text, each network was trained using 100,000 or more simulated data sets, created by drawing parameter values from the priors and using them to simulate responses to the stimuli presented to one of our participants. The simulated data were then split into training and validation sets, composed of 70% and 30% of the simulated data, respectively. This allowed us to accurately check cross validation accuracy during and after training, while still giving the neural networks enough data for a smooth gradient.

The neural network was trained using the ADAM algorithm (Kingma & Ba, 2014), which uses an adaptive learning rate mechanism to rapidly converge on global minima, or the best predictions for model parameters from the data. We ran this algorithm for 2500 epochs, which was sufficient to converge – by reaching a plateau in prediction accuracy – for all models examined here. The loss function for the neural network was mean squared error, allowing the neural network to treat parameter estimation as a regression problem. This naturally gives slightly greater weight to parameters that varied more widely; for this reason, we tried to use prior distributions of parameters with similar variance when creating the simulated data sets.

Once the neural network was trained, we evaluated its performance on each simulated data set in the validation set, generating 30,000 or more predictions from each network. These predictions were then compared against the true parameters used to create each of the validation data sets, giving us a metric for comparing network performance against ground-truth values in a cross-validation test.

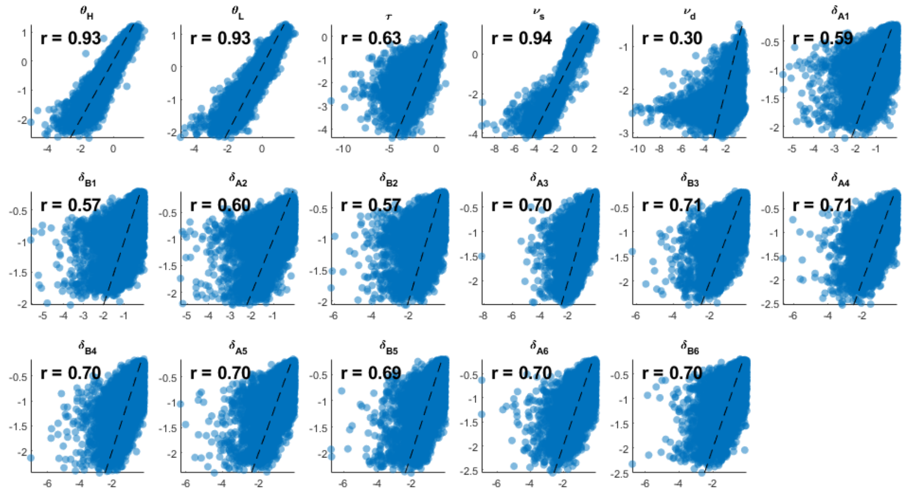

Figure S2

Parameter recovery study for the racing accumulator model. Each subplot displays a scatterplot of 1000 true (x) versus estimated (y) values of a different parameter of a stochastic accumulator model with absolute-evidence stopping rule. To ensure that all values are positive, all parameters have been log-transformed for estimation. The parameter θ *corresponds to thresholds (2),* τ *to non-decision time,* ν_s *to start point variability,* ν_δ *to drift rate variability, and* δ *parameters correspond to the drift rates for each of the twelve conditions in the study.*

To match the number of drift parameters across the models, we fixed the drift direction based on the match manipulation. Specifically, the drift rate for the "correct" accumulator was proportional to the average proportion of draws from the stimulus that would be more consistent with the correct response than the incorrect response, and the drift rate for the "incorrect" accumulator was proportional to the average percentage of draws from the stimulus that would be expected to be closer to the incorrect response. This was calculated by taking the cumulative density of the wrapped normal distribution used to generate stimulus orientations above and below the zero-point (i.e., the orientation that was perfectly in between the two options). For example, if the correct option A was at 15 degrees, the incorrect option B was at -15 degrees, the stimulus mean was 5 degrees, and the stimulus standard deviation was 10 degrees, we would expect $\Phi(-5, \sigma = 10) = 31\%$ of samples to favor option B / incorrect and 69% of samples to favor option A / correct. These values were multiplied by a free parameter corresponding to drift magnitude, which scaled the two proportions by a constant to obtain the overall mean drift for each option. The overall drift rate for either accumulator was then calculated by adding a normal random variable (drift rate variability), sampled independently on every trial and for each accumulator, to this mean drift.

The results for the diffusion and accumulator models are shown in Figures S1 and S2, respectively. In general, the neural networks performed well when recovering thresholds, drift rates, non-decision times, and start point variability. They performed slightly worse at recovering drift rate variability, although this was not a parameter of particular interest. Based on these parameter recovery studies, for both models, we should expect them to yield reasonable and precise parameter values for the real data.

Note that we did not estimate the posterior variance of these parameters as in other modeling applications (Radev et al., 2020; Sokratous et al., 2023), simply because the precision of parameter estimation on the real data sets was not of particular interest.

Once parameter estimates were obtained for each of the participants in the real experiment, we created posterior predictions from the model by creating one more simulated data set based on the parameter estimates. This allowed us to create a simulated data set, based on the best predictions from the neural network, that mimicked perfectly the structure of real data. The mean and standard error of this simulated data set are shown in the main text for the accumulator, diffusion, and GSR models, and for two additional models (the LCA and LBA) below.

Additional models

There are by this point in time many models of two-alternative forced-choice tasks, many of which are covered in reviews by Ratcliff et al. (2016) and Busemeyer et al. (2019). Our goal in the main text was to examine the relationship between relative-evidence / diffusion models and absolute-evidence / accumulator models, rather than to provide a comprehensive coverage of all models of binary choice. However, it is natural to ask question about several other popular choice models and the mechanisms they propose. We focus on two well-established models here: the leaky competing accumulator (LCA), which proposes interactions between accumulators in the form of lateral inhibition as well as leakage within each accumulator; and the linear ballistic accumulator (LBA), which removes within-trial variability in the evidence accumulation process.

For the time being, we do not visit models with complex multi-attribute or multi-accumulator structures (Diederich & Busemeyer, 2006; Roe et al., 2001; Hawkins & Heathcote, 2021), collapsing choice boundaries or urgency gating (Hawkins et al., 2015; Drugowitsch et al., 2012), serial order accumulation models (Holmes et al., 2016; Diederich & Trueblood, 2018), or other models with leakage or other self-modulating parameters (Heath, 2000; Busemeyer & Townsend, 1993). Each of these types of models is a potential target for future work devoted to evaluating the importance of these different mechanisms. The machine learning approach to model estimation and comparison is likely to be fruitful with many of them as well. Yet the scope of such an endeavor would likely balloon this paper to an enormous size, and detract from the message(s) focusing on accumulator and diffusion models.

Leaky competing accumulator

A natural question to ask is how competition among options, or decay in the support for available options, impacts choice (Teodorescu & Usher, 2013). As experimenters or modelers, we do not have direct access to the degree of support that a participant has for each available option. In diffusion and independent accumulator models, this is not a particularly large concern, as the degree of support for each option is directly related to the evidence under consideration (plus some bias or noise). However, in cases where the degree of support for an option depends not only on how much support it already has (leakage) or on how much support other options have (lateral inhibition), then we cannot infer the degree of support from the information that is presented to each participant. This makes deriving accumulated evidence profiles for models like the leaky competing accumulator (Usher & McClelland, 2001, 2004) a more complex problem.

Fortunately, we can simulate the evidence accumulation process with decay and lateral inhibition to understand its predictions (Turner et al., 2016). To derive an accumulated evidence

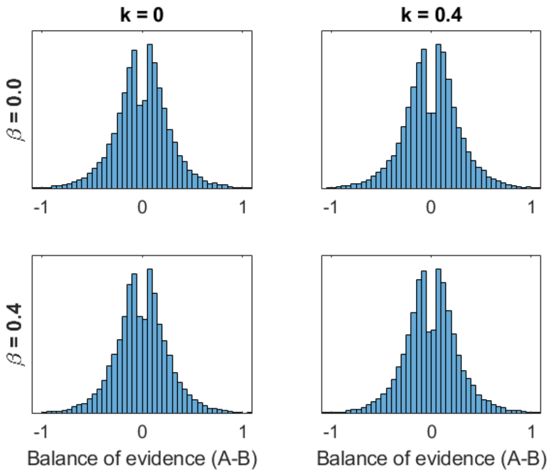

Figure S3

Distributions of evidence that participants might collect when making decisions under the leaky competing accumulator model. The top row shows show decisions made with low levels of lateral inhibition , while the bottom row shows decisions made under high levels of lateral inhibition (β). Left panels show decisions under low levels of leakage, while right panels show decisions under high levels of leakage (k)

profile, we simply simulate many trials from the model and record the stimulus information that a (simulated) participant obtains en route to hitting a threshold and terminating the trial. This allows us to make inferences about what distributions of stimulus information we might observe if someone were using a leaky competing accumulation process to make their decisions.

The resulting patterns of stimulus information that participants might consider when following an LCA decision process is shown in Figure S3. In general, this model tends to resemble the diffusion model in that it has a “gap” in the middle of stimulus evidence. We suspect this is for a similar underlying reason – that moderate information does not tend to terminate search 00 although it may be attributable to a slightly different mechanism in the LCA. Specifically, in the LCA, an accumulator has to overcome both the degree of support for the other option (lateral inhibition) and its own upper limits on the amount of evidence that can be accumulated for an individual option (leakage). It also tends to be heavily influenced by early samples of evidence, such that a large advantage for one model over another early in the evidence accumulation process can lead to particularly quick decisions in favor of that option – as it is not being inhibited by the other option and not subject to as much leakage.

As a result, extreme samples of evidence can have an outsized effect in the LCA. They tend to give one accumulator an early advantage, create lateral inhibition against the trailing option, and circumvent the leakage that occurs when evidence accumulates slowly. As a result, the evidence accumulation process will tend to halt more often after gathering an extreme piece of evidence that strongly favors one option compared to after gathering a moderate piece of evidence that does not provide much support for one option over the other. We suspect that this ultimately leads to the bimodal distribution of stimulus evidence observed in Figure S3, where extreme information is oversampled relative to moderate information.

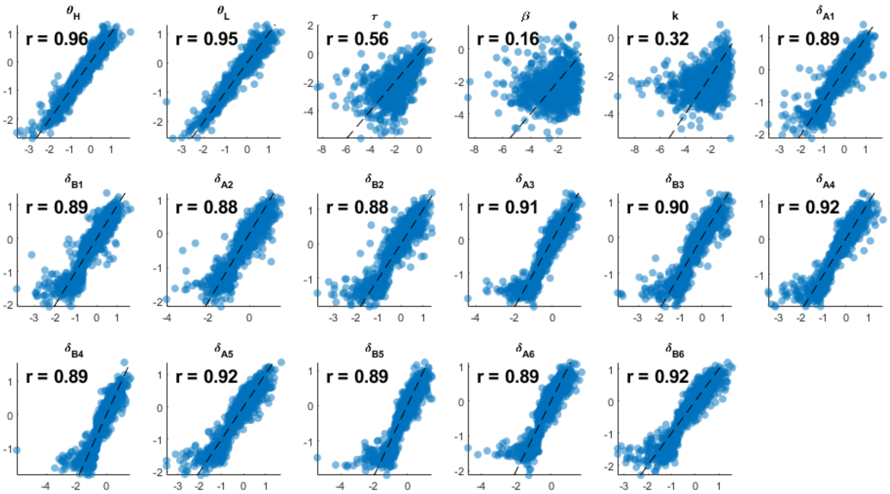

Figure S4

Parameter recovery study for the leaky competing accumulator model. Each subplot displays a scatterplot of 1000 true (x) versus estimated (y) values of a different parameter of the LCA. To ensure that all values are positive, all parameters have been log-transformed for estimation. The parameter θ *corresponds to thresholds (2),* τ *to non-decision time,* β *to lateral inhibition, k to leakage, and* δ *corresponds to the drift rates for each of the twelve conditions in the study.*

Parameter estimation

The leaky competing accumulator model is an early example of a model that does not have a tractable likelihood function (Usher & McClelland, 2001). As a result, it requires simulation-based methods to fit (Turner et al., 2016). This makes it ideally suited for estimation using machine learning methods like amortized inference (Radev et al., 2020; Sokratous et al., 2023; Rmus et al., 2023). Here, we present a parameter recovery study for the LCA using this approach. It has previously been validated using other methods (Miletić et al., 2017), but doing so here allows us to also fit it to the data presented in the main text.

The LCA we tested used the same number of parameter as the accumulator and diffusion models presented above. It swapped the across-trial variability parameters, drift rate variability and start point variability, for leakage k and lateral inhibition β. All other parameters were the same, including the 12 different drift rates for the 12 different conditions. The drift direction was fixed based on the match manipulation, allowing us to estimate only a single drift parameter for the two accumulators and limit the number of free parameters in the model to something comparable to the diffusion model.

The priors for the parameters were the same as those used for the accumulator model, with the exception of the leakage and lateral inhibition parameters. Each of these parameters was drawn from a beta distribution $B(1,5)$, which resulted in values for these parameters that were grouped mostly close to zero and bounded between zero and one. This reflects roughly the real values that are ordinarily obtained for these parameters in other studies (Usher & McClelland, 2001, 2004; Miletić et al., 2017). As before, all parameters were log-transformed for network training and

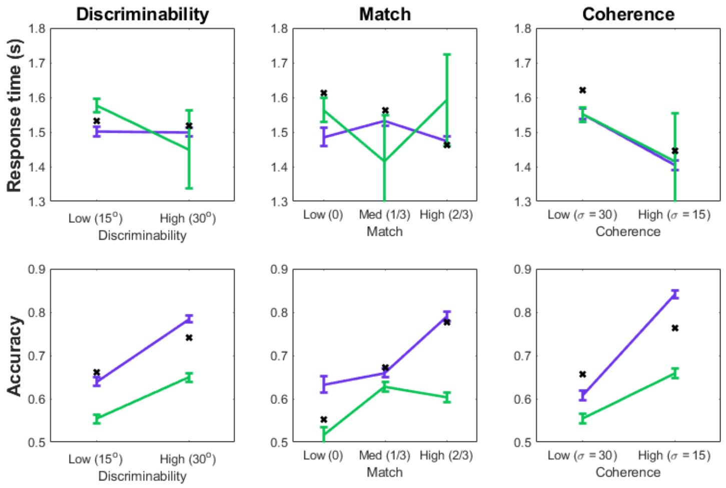

Figure S5

Posterior predictions from the linear ballistic accumulator (green) and leaky competing accumulator model (purple) for the patterns of mean response times (top) and accuracy (bottom), plotted against the data (black x). Error bars correspond to the predicted standard error of the mean from a single simulated data set generated by the best-fit parameters of each model.

parameter estimation, to ensure that they were all positive.

The results of this parameter estimation method are shown in Figure S4. Threshold (θ_{1-2}) and drift rate (δ_{1-12}) parameters showed excellent recovery. Non-decision time (τ) and leakage (k) showed reasonable recovery, while lateral inhibition was particularly difficult to estimate. As a result, the leaky competing accumulator model as a whole shows fairly good parameter recovery, but the parameters that most distinguish it from other accumulator models – leakage and lateral inhibition – are the most difficult to estimate. It is not entirely clear that its predictions will differ much from those of the accumulator model covered in the main text.

In addition to parameter recovery, we also used the resulting networks to fit the leaky competing accumulator model to the empirical data from the experiment reviewed in the main text. The resulting predictions for mean response time and accuracy are shown in Figure S5 in purple.

In general, the LCA shows a pattern of model fits that is quite similar to the accumulator model in the main text. It reproduces the most important phenomena – accuracy that increases with discriminability, match, and coherence as well as response times that decrease with match and coherence but not discriminability. In general, the LCA is a plausible model with regard to the data and it would be difficult to rule out based on the fits to this data set. The catch here is that its predictions are almost identical to that of the accumulator model, and without reliable estimation of decay and leakage parameters, it would be hard to tell the difference between the two.

Linear ballistic accumulator

In addition assumptions related to leakage and lateral inhibition, we also examined the assumption that evidence in the accumulator model is accumulated stochastically. The linear ballistic accumulator model [LBA] (Brown & Heathcote, 2008) provides an account of major phenomena in binary choice – including the speed-accuracy trade-off, fast and slow errors, and the shape of

response time distributions – without the need for within-trial noise in the evidence accumulation process. It constitutes a substantial simplification on the stochastic accumulator models, both from a mathematical and computational standpoint. As a result, simulating and fitting this model is substantially quicker than simulating and fitting the accumulator model used in the main text.

Although it captures the major phenomena in binary choice, is is still important to ensure that the LBA can be fit to data and that it can capture how accuracy and response times response to manipulations. Assuming no within-trial noise comes with the caveat that the LBA has no sensible random walk implementation. This means that the accumulated-evidence profiles make very little sense from the perspective of the LBA, as the initial piece of evidence entirely drives the accumulation process. One option is to look at the balance of evidence between the accumulators at the conclusion of a trial rather than the stimulus information that is provided between the start and end of a trial; this is done in the supplementary materials of Kvam et al. (2022). If we look at accumulator evidence rather than stimulus information, the balance of evidence between choice options at the end of an LBA process looks very similar to the balance of evidence predicted by the accumulator model examined in the main text.

Accumulated evidence profiles tend not to tell us much about the LBA, but we can still look at its performance in terms of recreating the patterns of accuracy and response time in the experimental data. To match it with the other models we examined, we trained a neural network to map observed data onto the parameters of the LBA as before. The number of simulations, training epochs, network structure, activation functions, hidden layers, training-validation split, and even number of parameters were the same as the accumulator model examined in the main text. As with the accumulator model, the threshold was permitted to vary with the discriminability manipulation and drift rates were permitted to vary across all combinations of coherence, match, and discriminability conditions. In addition to non-decision time, start point variability, and drift variability, this totaled 17 parameters – again, the same as the accumulator model from the main text.

The parameter recovery for the LBA is shown in Figure S6. The threshold and across-trial variability parameters were recovered reasonably well, similar to the other accumulator model. However, estimates of the drift rates in this model were relative poor by comparison, typically only corresponding with the true (log) drift rates with a correlation around .3. This is likely to result in a high degree of uncertainty in the LBA drift rate estimates, and potentially a high degree of variability from participant to participant and condition to condition when fit to the real data.

The performance of the LBA on the mean accuracy and response time data is shown in Figure S5. While it generally tends to follow the correct trends for each manipulation, the LBA generally under-estimated accuracy across conditions and showed a high degree of variability across participants and across trials. This is not too surprising given the unreliability of the parameter recovery study shown in Figure S6, and constitutes a potential problem for this version of the LBA. While we still consider it a plausible account of the phenomena, it is certainly outperformed by the LCA, accumulator, and GSR models on these data.

Like diffusion and accumulator models, neither the LBA nor the LCA really explain *why* the three different types of manipulations – discriminability, match, and coherence – create the patterns in accuracy and response times. All four models explain these phenomena in terms of changes in drift rates across the twelve conditions, combining these conceptually distinct manipulations into a single model parameter. In that sense, they lack explanatory utility when compared to the GSR, which attributes changes in the three manipulations to different elements of the accumulation process. In this new approach, discriminability corresponds to the angles between options, match to

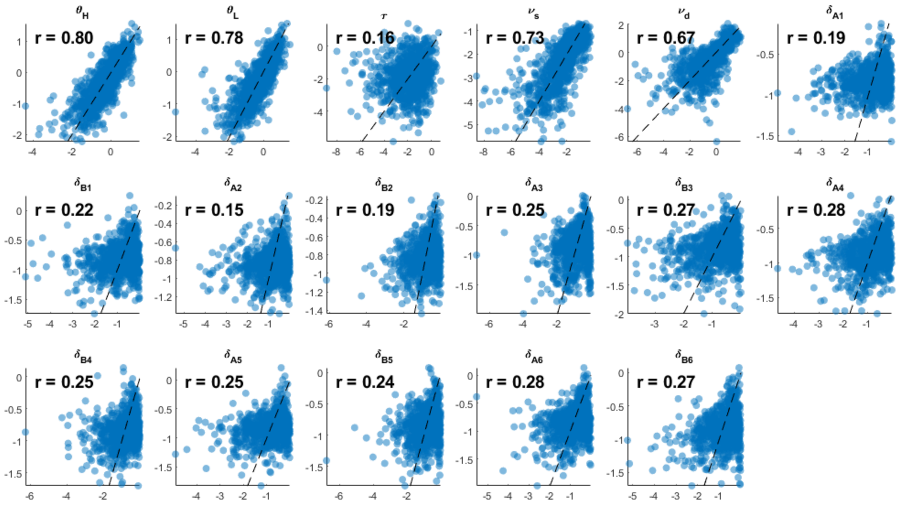

Figure S6

Parameter recovery study for the linear ballistic accumulator model. Each subplot displays a scatterplot of 1000 true (x) versus estimated (y) values of a different parameter of a stochastic accumulator model with absolute-evidence stopping rule. To ensure that all values are positive, all parameters have been log-transformed for estimation. The parameter θ corresponds to thresholds (2), τ to non-decision time, ν_s to start point variability, ν_δ to drift rate variability, and δ parameters correspond to the drift rates for each of the twelve conditions in the study.

the drift direction, and coherence to the drift magnitude – three different elements of the model. This is both conceptually and parsimoniously advantageous, as the GSR explains the pattern of behavior just as well with far fewer parameters, needing only 4 additional parameters (one more level of option direction γ, one more drift magnitude $|\delta|$, and two more drift directions ϕ_{2-3}) to explain 11 additional conditions.

Overall model comparison

Across the main text and supplement, we have covered a total of five models: the diffusion decision model, an accumulator model with within-trial noise, the geometric similarity representation model, the linear ballistic accumulator model, and the leaky competing accumulator model. With data simulated from all five models for the task shown in the main text, it is relatively straightforward to train a neural network to discriminate among all of them. We therefore implemented a neural network aimed at classifying an input data set based on which of the five models was used to generate it.

To train the network, we used the same method described above. Data from each model was summarized using the 84 inputs (mean RTs for correct / incorrect, accuracy, and RT quantiles for each of the 12 conditions), fed into a neural network with 4 hidden layers, and connected to an output with five category probabilities. We generated 100,000 simulated participants from each of the five models, and split these into 70,000 that were used to train the network and 30,000 that were used to validate it. The network was trained using the ADAM algorithm across 5000 epochs. The

		Inferred model				
		Diffusion	Accumulator	GSR	LBA	LCA
	Diffusion	.9737	.0141	.0063	0	.0058
	Accumulator	.0210	.8242	.0309	.1065	.0175
True model	GSR	.0390	.1057	.8386	.0016	.0151
	LBA	0	.1302	.0005	.8609	.0084
	LCA	.0110	.009	.0060	0	.9740

Table S1

Model recovery by the classification network. Rows correspond to the model that was used to generate the data, and the columns correspond to the model that was identified as the most likely generative model by the network.

confusion matrix for this network is shown in Table S1.

As one might expect, it was particularly difficult for the network to tell the difference between the linear ballistic accumulator and the accumulator model that included within-trial noise. These two models are identical except for the inclusion of the random element to evidence accumulation that is present in the accumulator model used in the main text. As a result, the LBA and accumulator models had somewhat lower recovery accuracy than other models. Likewise, the GSR could be mistaken for accumulator or diffusion models in many cases by virtue of its option-B orientation parameter being close to π (diffusion) or $\pi/2$ (accumulator). Otherwise, the network tended to recover the generative model quite well, with an overall accuracy of 89.43%.

The resulting network was then applied to the real data. Each participant's data was again summarized using 84 summary statistics across the 12 conditions, and passed as inputs into the same network that had been trained based on the simulated data. The output of the network was 5 probabilities, corresponding to the posterior likelihood of each model being the one that generated the data. Overall, the diffusion model fit best for 4 participants (overall posterior probability = .11), the accumulator model fit best for 12 participants (overall posterior probability = .41), the GSR model fit best for 10 participants (overall posterior probability = .26), the LBA fit best for 5 participants (overall posterior probability = .18), and the LCA only fit best for 3 participants (overall posterior probability = .04).

The split between accumulator and GSR models is likely due to the estimates of γ in the GSR being quite close to $\pi/2$ in most cases. When this occurs, the GSR is identical to the accumulator model – and in fact the accumulator models are more flexible, because they allow for separate drift rates for each of the 12 conditions. As a result, the more complex accumulator-based models tended to have high posterior probabilities while the diffusion model had a quite low posterior probability.

It worth noting that the LCA and GSR had relatively wide priors on their unique parameters: the γ parameter in the GSR varied between 0 and π, and the leakage and lateral inhibition parameters were quite wide in the LCA. The classification approach is quite sensitive to these priors, as it assigns posterior probabilities based on how closely they match to all the sets of simulated data that model produces, as opposed to how closely they match only the best-fitting set of simulated data. Future work using the classification approach should explore how to make it more robust to variability in priors and simulated data to alleviate this problem. For example, it might consider using the posterior parameter estimates from a parameter estimation network to generate new train-

ing data from each model to use in the classification network, rather than re-using the same training data from the parameter estimation network.

References

Brown, S. D., & Heathcote, A. (2008). The simplest complete model of choice response time: Linear ballistic accumulation. *Cognitive Psychology*, *57*(3), 153–178. doi: 10.1016/j.cogpsych.2007.12.002

Busemeyer, J. R., Gluth, S., Rieskamp, J., & Turner, B. M. (2019). Cognitive and neural bases of multi-attribute, multi-alternative, value-based decisions. *Trends in Cognitive Sciences*, *23*(3), 251–263.

Busemeyer, J. R., & Townsend, J. T. (1993). Decision field theory: A dynamic-cognitive approach to decision making in an uncertain environment. *Psychological Review*, *100*(3), 432–459.

Diederich, A., & Busemeyer, J. R. (2006). Modeling the effects of payoff on response bias in a perceptual discrimination task: Bound-change, drift-rate-change, or two-stage-processing hypothesis. *Perception & Psychophysics*, *68*(2), 194–207. doi: 10.3758/BF03193669

Diederich, A., & Trueblood, J. S. (2018). A dynamic dual process model of risky decision making. *Psychological Review*, *125*(2), 270–292.

Drugowitsch, J., Moreno-Bote, R., Churchland, A. K., Shadlen, M. N., & Pouget, A. (2012). The cost of accumulating evidence in perceptual decision making. *The Journal of Neuroscience*, *32*(11), 3612–3628. doi: 10.1523/JNEUROSCI.4010-11.2012

Hawkins, G. E., Forstmann, B. U., Wagenmakers, E.-J., Ratcliff, R., & Brown, S. D. (2015). Revisiting the evidence for collapsing boundaries and urgency signals in perceptual decision-making. *The Journal of Neuroscience*, *35*(6), 2476–2484.

Hawkins, G. E., & Heathcote, A. (2021). Racing against the clock: Evidence-based versus time-based decisions. *Psychological Review*, *128*(2), 222–263.

Heath, R. A. (2000). The Ornstein-Uhlenbeck model for decision time in cognitive tasks: An example of control of nonlinear network dynamics. *Psychological Research*, *63*(2), 183–191.

Holmes, W. R., Trueblood, J. S., & Heathcote, A. (2016). A new framework for modeling decisions about changing information: The piecewise linear ballistic accumulator model. *Cognitive Psychology*, *85*, 1–29.

Kingma, D. P., & Ba, J. (2014). *Adam: A method for stochastic optimization.*

Kvam, P. D., Alaukik, A., Mims, C. E., Martemyanova, A., & Baldwin, M. (2022). Rational inference strategies and the genesis of polarization and extremism. *Scientific Reports*, *12*(1), 1–13.

Miletić, S., Turner, B. M., Forstmann, B. U., & van Maanen, L. (2017). Parameter recovery for the leaky competing accumulator model. *Journal of Mathematical Psychology*, *76*, 25–50.

Radev, S. T., Mertens, U. K., Voss, A., & Köthe, U. (2020). Towards end-to-end likelihood-free inference with convolutional neural networks. *British Journal of Mathematical and Statistical Psychology*, *73*(1), 23–43.

Ratcliff, R., Smith, P. L., Brown, S. D., & McKoon, G. (2016). Diffusion decision model: Current issues and history. *Trends in Cognitive Sciences*, *20*(4), 260–281.

Rmus, M., Pan, T.-F., Xia, L., & Collins, A. G. (2023). Artificial neural networks for model identification and parameter estimation in computational cognitive models. *Biorxiv*.

Roe, R. M., Busemeyer, J. R., & Townsend, J. T. (2001). Multialternative decision field theory: A dynamic connectionst model of decision making. *Psychological Review*, *108*(2), 370–392. doi: 10.1037/0033-295X.108.2.370

Sokratous, K., Fitch, A., & Kvam, P. D. (2023). How to ask twenty questions and win: Machine learning tools for assessing preferences from small samples of willingness-to-pay prices. *Journal of Choice Modelling*, *48*, 100418.

Teodorescu, A. R., & Usher, M. (2013). Disentangling decision models: from independence to competition. *Psychological Review*, *120*(1), 1.

Trueblood, J. S. (2022). Theories of context effects in multialternative, multiattribute choice. *Current Directions in Psychological Science*, *31*(5), 428–435.

Trueblood, J. S., Brown, S. D., & Heathcote, A. (2014). The multiattribute linear ballistic accumulator model of context effects in multialternative choice. *Psychological Review*, *121*(2), 179–205. doi: 10.1037/a0036137

Turner, B. M., Schley, D. R., Muller, C., & Tsetsos, K. (2018). Competing theories of multialternative, multiattribute preferential choice. *Psychological Review*, *125*(3), 329.

Turner, B. M., Sederberg, P. B., & McClelland, J. L. (2016). Bayesian analysis of simulation-based models. *Journal of Mathematical Psychology*, *72*, 191–199.

Usher, M., & McClelland, J. L. (2001). The time course of perceptual choice: The leaky, competing accumulator model. *Psychological Review*, *108*(3), 550–592. doi: 10.1037/0033-295X.108.3.550

Usher, M., & McClelland, J. L. (2004). Loss aversion and inhibition in dynamical models of multialternative choice. *Psychological Review*, *111*(3), 757–769. doi: 10.1037/0033-295X.111.3.757
